# Actively transcribed rDNA and distal junction (DJ) sequence are involved in association of NORs with nucleoli

**DOI:** 10.1007/s00018-023-04770-3

**Published:** 2023-04-12

**Authors:** Mikhail Liskovykh, Nikolai S. Petrov, Vladimir N. Noskov, Hiroshi Masumoto, William C. Earnshaw, David Schlessinger, Svetlana A. Shabalina, Vladimir Larionov, Natalay Kouprina

**Affiliations:** 1 Developmental Therapeutics Branch, National Cancer Institute, NIH, Bethesda, MD 20892 USA; 2 Laboratory of Chromosome Engineering, Department of Frontier Research and Development, Kazusa DNA Research Institute, Kisarazu, Chiba 292-0818 Japan; 3 Wellcome Centre for Cell Biology, University of Edinburgh, Edinburgh, EH9 3JR Scotland, UK; 4 National Institute on Aging, Laboratory of Genetics and Genomics, NIH, Baltimore, MD 21224 USA; 5 National Center for Biotechnology Information, National Library of Medicine, NIH, Bethesda, MD 20892 USA

**Keywords:** rDNA, Ribosomal DNA, Nucleolar organizer regions (NORs), Human artificial chromosome (HAC), Transformation-associated recombination (TAR)

## Abstract

Although they are organelles without a limiting membrane, nucleoli have an exclusive structure, built upon the rDNA-rich acrocentric short arms of five human chromosomes (nucleolar organizer regions or NORs). This has raised the question: what are the structural features of a chromosome required for its inclusion in a nucleolus? Previous work has suggested that sequences adjacent to the tandemly repeated rDNA repeat units (DJ, distal junction sequence) may be involved, and we have extended such studies by addressing several issues related to the requirements for the association of NORs with nucleoli. We exploited both a set of somatic cell hybrids containing individual human acrocentric chromosomes and a set of Human Artificial Chromosomes (HACs) carrying different parts of a NOR, including an rDNA unit or DJ or PJ (proximal junction) sequence. Association of NORs with nucleoli was increased when constituent rDNA was transcribed and may be also affected by the status of heterochromatin blocks formed next to the rDNA arrays. Furthermore, our data suggest that a relatively small size DJ region, highly conserved in evolution, is also involved, along with the rDNA repeats, in the localization of p-arms of acrocentric chromosomes in nucleoli. Thus, we infer a cooperative action of rDNA sequence—stimulated by its activity—and sequences distal to rDNA contributing to incorporation into nucleoli. Analysis of NOR sequences also identified LncRNA_038958 in the DJ, a candidate transcript with the region of the suggested promoter that is located close to the DJ/rDNA boundary and contains CTCF binding sites. This LncRNA may affect RNA Polymerase I and/or nucleolar activity. Our findings provide the basis for future studies to determine which RNAs and proteins interact critically with NOR sequences to organize the higher-order structure of nucleoli and their function in normal cells and pathological states.

**Supplementary Information:**

The online version contains supplementary material available at 10.1007/s00018-023-04770-3.

## Introduction

**Fig. 1:**
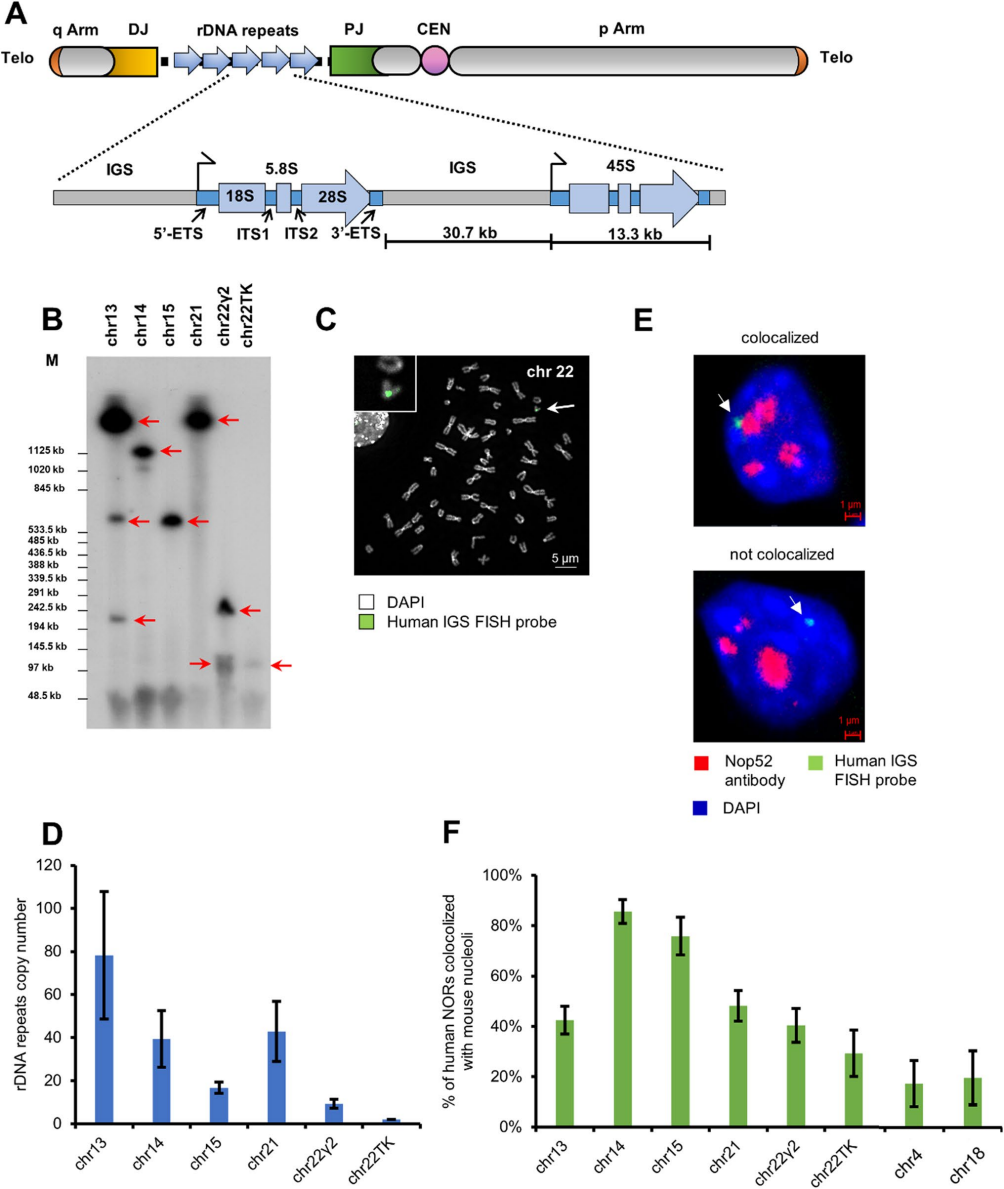
**a** Schematic representation of the acrocentric human chromosomes. Scheme of an acrocentric chromosome with the rDNA portion and distal (DJ) and proximal (PJ) junction sequences. rDNA repeats are located on q Arm of the chromosome. The number of rDNA repeats varies on the chromosomes. Each ~ 13.3 kb rDNA repeat is composed of the transcribed region encoding 45S rRNA (5′-ETS, 18S, ITS1, 5.8S, ITS2, 28S and 3′-ETS) and a ~ 30.7 kb IGS (intergenic spacer sequence). The 13.3 kb transcribed human 45S rDNA region is shown in blue. Telo-telomere regions. CEN-centromere region. **b** Southern blot analysis of six human/mouse hybrid cell lines containing human chromosomes 13, 14, 15, 21 and 22 [A9 (#13) 89-2 (chr13), A9 hygro14 10F (chr14), A9 (Neo15)-3 (chr15), A9 #21-16 (chr21), A9#22 (γ2) (chr22γ2) and A9HyTK-22 (chr22TK)]. Genomic DNA possessing one of the acrocentric chromosomes was isolated from each hybrid cell line, digested by EcoRV and separated by CHEF gel electrophoresis (range 50 kb–1.1 Mb). The rDNA repeats were detected with a radioactively labeled probe specific to the rDNA intergenic spacer (IGS). Red arrowheads indicate the fragments that contain rDNA repeats. *M1* a marker [CHEF DNA Size Lambda Ladder (BIO-RAD)]. **c** FISH analysis of human/mouse hybrid cell line A9#22 (γ2) containing chromosome 22 with the human IGS FISH probe specific to the intergenic spacer of the rDNA unit (see “Materials and methods”). Chromosome 22 (green) is indicated by a white arrowhead. **d** A copy number of the rDNA repeats in a human acrocentric chromosome in each hybrid cell line was estimated by quantitative real-time PCR (qPCR). **e** 3D immuno-FISH was performed on human/mouse hybrid cells. Anti-RRP1 antibodies (Nop52) (red) used to visualize nucleoli were combined with a BAC FISH probe containing the IGS sequence (green). Nuclei were stained with DAPI (blue). Nucleolar- and non-nucleolar-associated NORs are indicated by white arrowheads. **f** Quantification of 3D immuno-FISH showing the percentage of cells in which the NORs from human acrocentric chromosomes and human non-acrocentric chromosomes 4 and 18 associated with mouse nucleoli. For each cell line, the experiment was replicated three times with the total number of the nucleolar localized and non-localized chromosomes ranged from 141 to 317 (Table S3)

Ribosomal RNA (rDNA) gene clusters occupy a significant part of the genome, forming repeat regions comparable to centromeres. The total copy number of rDNA repeats varies from 250 to 670 per diploid human genome [1]. The repeats are generally organized as tandem arrays located on the short arms of the five acrocentric chromosomes 13, 14, 15, 21, and 22. rDNA arrays are the main components of nucleolar organizer regions (NORs). The number of rDNA repeats in individual human NORs ranges from 1–2 (~ 50–100 kb) to more than 130 (~ 6 Mb) [2–4]. Each repeat contains the coding sequence for a ~ 13.3 kb pre-rRNA comprising a copy of 18S, 5.8S, and 28S rRNA sequences separated by internal transcribed spacer sequences (ITSs) and flanked by external transcribed spacers (ETSs) (5′-ETS, 18S, ITS1, 5.8S, ITS2, 28S, 3′-ETS). The transcribed portion is followed by an ~ 30.7 kb non-transcribed intergenic spacer (IGS) (Fig. 1a). The IGS houses gene promoters and regulatory elements such as repetitive enhancer elements that control pre-rRNA synthesis [5]. The IGS also contains replication origins and replication fork barriers (RFBs) that prevent collisions between the replication and transcription machineries [6–8].

The primary 45S pre-rRNA transcript is transcribed from rDNA units by RNA polymerase I (RNA Pol I) and is then processed to produce the mature 18S, 5.8S and 28S RNAs (rRNA) [9, 10] that participate in the assembly of ribosomes. In most human cells, only 20–50% of all RNA genes are transcriptionally active [11, 12]. Active rDNA units are bound to the upstream binding factor (UBF) and form the NORs [13–15]. Nucleoli, which are the sites of ribosome biogenesis and the largest substructures in eukaryotic nuclei, form around arrays of rDNA. Some inactive rDNA copies are located at the periphery of the nucleolus and shape so-called perinucleolar heterochromatin, while actively transcribed rDNA repeats are positioned within the interior of the nucleolus [16, 17].

The apparent regularity of rDNA repeats is consistent with the notion that each ribosome has been considered equivalent to all others. The notion of ribosome uniformity was further reinforced when a 3D structure of ribosomes was established at high resolution [18] and a reference sequence of a ~ 45 kb rDNA unit encoding the 45S rRNA precursor was assembled (U13369.1, GenBank). Nevertheless, for a long time, the long-range analysis of rDNA regions of the acrocentric chromosomes has remained little investigated, because of the tendency of rDNA-containing clones to rearrange and delete contents [19] and because rDNA sequences coming from different chromosomes can create ambiguous sequence assemblies.

The organization and sequence variation of human rDNA units is likely to be highly significant, both because NORs are the locus of ribosome biogenesis and because they have also been implicated in processes as varied as cellular stress response, mitosis and cell proliferation, and specific age-related pathology (summarized in [5]).

Given the inferred involvement of NORs in so many important processes, we have recently aimed to characterize the actual structure and variation in human NORs. Using Transformation-Associated Recombination (TAR) cloning [20, 21], we isolated the ~ 820 kb rDNA region of chromosome 21 from the A9 (21–16) cell line [22] and the ~ 240 kb entire NOR, containing the rDNA complement and proximal (centromeric) and distal (telomeric) junctions [20] that might be involved in nucleolar function, of a chromosome 22 from the A9HyTK-22 cell line [23]. TAR cloning of 18 of ~ 56 copies of the rDNA unit from chromosome 21 provided a preliminary scaffold for much of one acrocentric chromosomal arm and provided reagents for the further mapping of the NOR [22]. In contrast to chromosome 21, there are only two copies of rDNA units in the NOR of chromosome 22 from the A9HyTK-22 cell line. (We note that the rDNA copy numbers for these acrocentric chromosomes refer to our experimental material; copy numbers in individuals vary across human populations).

Sequence analysis of rDNA isolates from the sampled chromosome 21 revealed an unexpectedly high level of heterogeneity in human rDNA, including substantial base substitutions, variation in mono- or dinucleotide run lengths, large insertions, and deletions [22]. The rDNA TAR clones contained 101 variant positions in the 45S transcription unit and 235 in the intergenic spacer sequence. This raises the possibility to investigate the role of rDNA variation in nucleolar formation, ribosome biogenesis, and possible associations with pathology. Variants detected from rDNA isolates from chromosome 21 also facilitated annotation of a new, high-quality 44,838 bp rDNA reference sequence (KY962518 in GenBank) [22]. Recently a group has published the first complete sequence of the human genome that includes a sequence of the short arms of all five acrocentric chromosomes. The rDNA assembly contains 219 complete rDNA copies, totaling 9.9 Mb of a sequence [24]. The study confirmed our observations on a high level of variants in human rDNA units as well as in a new reference sequence.

The possible effects of variants in ribosomal DNA sequence have not yet been studied, but further investigation of the role of rDNA sequence in nucleolar formation as well as function is warranted by reports of various phenotypic effects of genome dosage and/or number of active rRNA genes in health and pathology. For example, it was recently suggested that rheumatic arthritis genomes appeared to harbor significantly less active ribosomal genes compared to controls [25]. Another study suggested that reduced ribosomal activity is a major contributor to the pathology of Alzheimer’s disease [26]; and it has been reported that Alzheimer’s disease is accompanied by epigenetic rDNA silencing, which reduces the number of active rRNA genes while the total number of ribosomal repeats is not decreased [27]. rDNA alterations have also been detected in many cancers, manifested as changes in nucleolar morphology and upregulation of rRNA transcription and ribosome biogenesis [28–30]. The size and/or number of nucleoli are increased in tumor cells, serving as an indicator of the rates of cell proliferation; this feature is a diagnostic marker for some cancers [31].

As for the sequences bracketing rDNA clusters, McStay et al. [32, 33] have initiated studies of both proximal and distal junctions (DJ and PJ). Starting from the sequences of BAC and cosmid clones deposited in GenBank, they first inferred 207 kb of sequence proximal and 379 kb of sequence distal to rDNA arrays [32]. They then further extended sequence analyses to infer distal junction contigs from individual human acrocentric chromosomes carried in human/mouse hybrid cells [33]. The DJ contigs from all five acrocentric chromosomes proved to be > 99% identical over ~ 300 kb and are ~ 90% identical over the following ~ 100 kb of a sequence. In particular, the junction point between the DJ sequence and the start of the first rDNA repeat unit lies ~ 4 kb 5′ of the first nucleotide in the 45S pre-rRNA transcript of rDNA and is 100% identical in representative sequence from all five acrocentric chromosomes. The same lab also showed that sequences proximal and distal to NORs are packaged in heterochromatin located at the nucleolar periphery [32]. Moreover, synthetic arrays of DJ sequences integrated into metacentric human chromosomes are also nucleolar-associated [33]. Based on these data, the authors concluded that rDNA arrays and DJ sequences are both physically and functionally linked and NORs should be considered as rDNA arrays and DJ sequences which may be involved in the establishment of NOR territories [33]. Moreover, based on their findings, they inferred that the association of acrocentric p-arms with the nucleoli is very likely driven by the activity or properties of the DJ sequences.

It was previously reported that the DJ region, rather than being a passive block of heterochromatin, is transcriptionally active [32]. Three transcripts were found within the DJ inverted repeat arms, with transcription originating from promoters at 138 kb, 187 kb and 238 kb in the DJ. The first transcript lies within the ACRO138 repeats. Two transcripts, termed as disnor187 and disnor238, may function as long noncoding RNAs (LncRNAs) [32]. These transcripts, when depleted, result in nucleolar stress, indicating their potential role in nucleolar function [33].

“DJ-like” sequences and the sequences of the specific transcripts from DJ DNA are conserved evolutionarily in chimpanzee (*Pan troglodytes*) compared to humans [33]. For example, transcripts disnor187 and disnor238 are 95.4 and 97.4% identical, respectively. NORs are located on the acrocentric chromosome arms of the same chromosomes—13, 14, 15, 21, and 22—in chimpanzee as well as in humans, with similar features ranging from inverted repeat arms all the way to the conservation of sequence ~ 96% identity over the first 300 kb of DJ sequence and ~ 80% identity thereafter [33]. By contrast, sequences that lie proximal to rDNA arrays (PJ) may contain elements that regulate NOR function but have remained poorly characterized, because these sequences are almost entirely segmentally duplicated, making the assembly of their sequence difficult.

In this work, we addressed several issues in relation to the requirements for the association of NORs with the nucleoli. The key question that we wish to answer was, what is the role of NOR context, which includes rDNA and DJ sequences, in nucleolar fusion? To answer this question, we exploited a panel of monochromosomal somatic cell hybrids containing an individual human acrocentric chromosome. As shown earlier, human rDNA is not transcribed in a mouse background, but human acrocentric chromosomes can associate with hybrid cell nucleoli [34, 35]. We also exploited a human artificial chromosome (HAC) carrying different parts of a NOR, including an rDNA unit or DJ or PJ region. The HAC contains characteristic chromosomal components, i.e., centromere, kinetochore, scaffold, and periphery, that make it capable of replicating and segregating like classical endogenous chromosomes in human cells [36–39]. In addition, the DNA insert in the HAC is in a chromatin domain that favors transcription and is bordered by heterochromatin segments of DNA that are required for proper centromere function [37, 39].

Herein, we report the localization of the transcriptionally silent ribosomal genes of human acrocentric chromosomes in hybrid cell nucleoli and show that the association of rDNA with nucleoli is not affected by the number of rDNA repeats but depends on the transcription of rDNA. Our data further suggest that other factors affect the association of NORs with nucleoli, i.e., heterochromatin blocks formed next to the rDNA arrays may participate, and a relatively small sequence of the DJ region, which is highly conserved in evolution, is itself enough to localize a HAC to nucleoli.

## Materials and methods

### Cell lines and media

Six human/mouse monochromosomal hybrid cell lines, each containing an individual acrocentric human chromosome, were used: A9 (#13) 89-2 (chromosome 13), A9 hygro14 10F (chromosome 14), A9 (Neo15)-3 (chromosome 15), A9 #21-16 (chromosome 21), A9#22 (γ2) (chromosome 22) and A9HyTK-22 (chromosome 22). Two human/mouse hybrid cell lines, A9KM10-4 and A9 48-4, containing non-acrocentric chromosome 4 and chromosome 18, respectively, were also used in our study. The cells were cultured in Dulbecco’s modified Eagle’s medium supplemented with 10% fetal bovine serum (Atlanta Biologicals, Lawrenceville, GA, USA) and 400 μg/ml Hygromycin B for A9HyTK-22 and A9 hygro14 10F and 800 μg/ml G418 (InvivoGen) for A9 (#13) 89-2, A9 (Neo15)-3, A9 #21-16 and A9#22 (γ2) at 37 °C in 5% CO_2_.

Hypoxanthine phosphoribosyltransferase (Hprt)-deficient Chinese hamster ovary (CHO) cells (JCRB0218) carrying the alphoid^tetO^-HAC were maintained in Ham’s F-12 nutrient mixture (Invitrogen, USA) plus 10% fetal bovine serum (FBS) with 8 μg/ml Blasticidin S Hydrochloride (Funakoshi, Japan). After insertion of the TAR BAC constructs into the alphoid^tetO^-HAC, CHO cells were maintained in the HAT-containing medium.

Human male fibrosarcoma HT1080 cell line (ATCC CCL 121) having a pseudodiploid karyotype and containing an activated N-ras oncogene was cultured in Dulbecco’s modified Eagle’s medium (DMEM) (Invitrogen) supplemented with 10% (v/v) tet system-approved fetal bovine serum (Clontech Laboratories, Inc.) at 37 °C in 5% CO_2_. HT1080 cells containing alphoid^tetO^-HAC were cultured in a medium containing Blasticidin (BS) at a concentration of 4 µg/ml to select cells containing the HAC carrying different TAR constructs. The HT1080 cells containing either alphoid^tetO^-HAC/tTA^VP64^ or alphoid^tetO^-HAC/tTS were cultured in 1 × HAT medium, 4 μg/ml Blasticidin S and 1 μg/ml doxycycline (Life Technologies).

### Transcription of rRNA genes

Total RNA was isolated from human/mouse monochromosomal hybrid cell lines and human RPE cells with the RNeasy Plus Mini Kit (Quiagen, cat. no 74134). 100 ng of RNA were used for cDNA synthesis with an iScript gDNA Clear cDNA Synthesis Kit (BioRad, cat. no. 1725034). qPCR reactions were run in triplicates on a CFX Connect Real-Time PCR Detection System in 20 μl volume using 0.2 μl of cDNA, SsoAdvanced Universal SYBR Green Supermix (BioRad, cat. no. 1725270) and human-specific 45S rRNA primers or human/mouse GAPDH primers (h45S F/h45S R and GAPDH F/GAPDH R, correspondingly) (Table S1).

### Quantitative real-time PCR

Human rDNA copy number was estimated with two pairs of primers for the human intergenic spacer (IGS) by quantitative real-time PCR using genomic DNA isolated from human/mouse monochromosomal hybrid cells. As an internal control for a single copy region, we used a pair of primers specific for the mouse chromosome 5 ACTB region (Mouse Chr5 F/Mouse Chr5 R). Sequences of the primers used in this experiment are listed in Table S1. A9HyTK-22 cell line served as a reference as it was known to contain two copies of the rDNA repeat [23].

### Southern blot hybridization analysis

Genomic DNA was prepared in agarose plugs (0.5 × 10^6^ cells per plug) and restriction digested with EcoRV in the buffer recommended by the manufacturer. The digested DNA was separated by CHEF (contour-clamped homogeneous electric field) gel electrophoresis (CHEF Mapper, Bio-Rad) (autoprogram, 50–1100 kb range, 16 h run or 250–2500 kb range, 48 h run), transferred to a nylon membrane (Amersham Hybond-N+), and blot-hybridized with a 421 bp probe specific for the human rDNA spacer sequence (IGS probe). The DNA sequence for the probe was amplified by PCR using the primers FOR-F/REV-R (Table S1). The blot was incubated for 2 h at 65 °C in pre-hybridization Church’s buffer (0.5 M Na-phosphate buffer containing 7% SDS and 100 µg/ml of salmon sperm DNA). The labeled probe was heat denatured in boiling water for 5 min and snap-cooled on ice. The probe was added to the hybridization buffer and allowed to hybridize overnight at 65 °C. The blots were then washed twice in 2 × SSC (300 mM NaCl, 30 mM sodium citrate, pH 7.0), 0.05% sodium dodecyl sulfate (SDS) for 10 min at room temperature, then twice in 2 × SSC, 0.05% SDS for 5 min at 60 °C, twice in 0.5 × SSC, 0.05% SDS for 5 min at 60 °C and twice in 0.25 × SSC, 0.05% SDS for 5 min at 60 °C. The blots were exposed to X-ray film for 24–72 h at − 80 °C to visualize labeled DNA band(s).

### Fluorescence in situ hybridization (FISH) analysis

To obtain metaphase chromosome spreads, cells were incubated in a growth medium with 0.05 μg/ml of Colcemid (Gibco) overnight. Medium was aspirated, and the plate was washed with 1 × PBS. Cells were treated with 0.25% Trypsin, washed off the plate with DMEM, pelleted and resuspended in 10 ml of 50 mM KCl hypotonic solution for 30 min at 37 °C. Cells were fixed by three washes of ice-cold fixative solution (75% acetic acid, 25% methanol). Between each wash, cells were pelleted by centrifugation at 500 rcf for 4 min at 4 °C. Metaphase cells were evenly spread on a microscope slide and the fixative solution evaporated over boiling water and allowed to age 2 days at room temperature.

To stain human rDNA-containing chromosomes, slides were denatured by 70% formamide/2 × SSC for 2 min at 72 °C. Samples were dehydrated through 70%, 90% and 100% ethanol series for 4 min each and left to air-dry. The probe was JH10-BAC (accession MF164270) containing a human IGS sequence from chromosome 21 labeled with Green 496 dUTP (#42831, Enzo) using a Nick translation DNA labeling system 2.0 (#GEN111-50, Enzo). 100 ng of the labeled probe (5 μl of labeling reaction) was combined with 2.5 μl Human Cot-1 DNA and 5 μl Herring Sperm DNA. One-tenth volume of 3 M sodium acetate, pH 5.5 was added and the DNA was precipitated by the addition of 2.5 volumes of 100% ethanol. The DNA pellet was washed with 70% ethanol, briefly dried, and resuspended in 25 μl Hybrisol VII by incubation at 37 °C with intermittent mixing. Next, the probe was denatured by incubation at 78 °C for 10 min and left at 37 °C for 30 min. The hybridization mix probe was applied to the samples and incubated at 37 °C overnight. Slides were washed with 0.4 × SSC, 0.3% Tween 20 for 2 min at 72 °C, briefly rinsed with 2 × SSC, 0.1% Tween 20 and air-dried in the dark. The samples were mounted with VectaShield mounting medium containing DAPI (Vector Labs). FISH analysis of human/mouse hybrid cell line A9#22 (γ2) containing chromosome 22 with the human IGS FISH probe specific to the intergenic spacer of the rDNA unit is shown in Fig. 1c.

To stain the HACs, slides were rehydrated with PBS for 15 min and fixed in 4% formaldehyde in 1 × PBS for 2 min, followed by three 5 min PBS washes and ethanol series (of 70%, 90% and 100%, correspondingly) dehydration. 1 μl of 10 μM stock of PNA (peptide nucleic acid) FITC-labeled probe for the alphoid^tetO^ array (FITC-OO-ACCACTCCCTATCAG) (Panagene, South Korea) was mixed with 20 μl of hybridization buffer (10 mM Tris–HCl, pH 7.4; 70% Formamide; 5% Dextran sulfate) and applied to the slide, followed by denaturation at 80 °C for 3 min. Slides were hybridized for 2 h at room temperature in the dark, then were washed twice in 70% formamide, 10 mM Tris pH 7.2, 0.1% BSA followed by three washes with 1 × TBS, 0.08% Tween-20. Slides were dehydrated gradually with a series of 70%, 90% and 100% ethanol washes and mounted with Vectashield Vibrance Antifade Mounting Medium containing DAPI. Slides were analyzed by fluorescence microscopy. Imaging was performed using a DeltaVision microscopy imaging system in the CRC/LRBGE Fluorescence Imaging Facility (NIH).

### 3D immuno-FISH

Cells were grown on collagen-coated slides until reaching 60–90% confluence. Slides were washed once for 5 min in PBS, incubated in 0.1% Triton X-100/PBS for 2 min to remove background GFP fluorescence and fixed in 4% paraformaldehyde/PBS for 10 min. Slides were then washed three times for 5 min in PBS and permeabilized in 0.5% saponin, 0.5% v/v Triton X-100 in PBS for 10 min. Finally, the slides were washed three times for 5 min in PBS and incubated in 20% glycerol/PBS for 2 h at room temperature and snap frozen in liquid nitrogen and stored at − 80 °C.

To stain human rDNA in human/mouse monochromosomal hybrids, slides were removed from the freezer, rinsed in PBS, depurinated in 0.1 N HCl for 5 min, washed once in 2 × SSC for 5 min, the preequilibrated by incubating in 50% formamide/2 × SSC for 15 min at 37 °C. Next, 25 ml of hybridization mix containing 100 ng of the human IGS probe (see above) was applied to the slide and nuclear DNA was denatured by incubation at 73 °C for 12 min. Then the slides were incubated at 37 °C in a humidity chamber for 48 h. After hybridization, slides were washed three times for 5 min each with 50% formamide/2 × SSC at 42 °C, then three times for 5 min each with 0.1 × SSC at 60 °C, then three times for 5 min each with PBS at room temperature.

Nucleoli were immunostained with anti-RRP1 antibodies (Nop52, a protein involved in pre-rRNA maturation [40]) (Novus, cat. no. NBP1-85338), diluted 1:100 in 1% BSA/PBS for 1 h. Slides were washed three times with PBS for 10 min each and stained with secondary antibodies Anti-rabbit IgG Fab2 Alexa Fluor 555 (Cell Signaling, cat. no. 4413S), diluted 1:500 in 1% BSA/PBS for 1 h. Then slides were washed three times with PBS for 10 min each and coverslips were mounted with Vectashield Vibrance Antifade Mounting Medium containing DAPI.

To visualize nucleoli and the HAC, anti-RRP1 antibodies were combined with a PNA probe for the alphoid^tetO^ array of the HAC. Then the slides were fixed, permeabilized and snap frozen as described above. Next, the slides were thawed, rinsed with PBS, dehydrated in 70%, 90%, and 100% ethanol and air-dried. Next, 1 μl of 10 μM stock of PNA FITC-labeled probe for alphoid^tetO^ array (FITC-OO-ACCACTCCCTATCAG) (Panagene, South Korea) was mixed with 20 μl of hybridization buffer (10 mM Tris–HCl, pH 7.4; 70% formamide; 5% Dextran sulfate) and applied to the slide, followed by denaturation at 80 °C for 5 min. The slides were incubated for 2 h at room temperature in the dark and then washed twice for 15 min in 70% formamide, 10 mM Tris pH 7.2, 0.1% BSA. Next, the slides were washed three times with PBS. Then the nucleolar protein RRP1 was immunostained as described above. All the sides were mounted with Vectashield Vibrance Antifade Mounting Medium containing DAPI (Vector Laboratories, cat. no. H-1800).

Images were acquired on LSM780 laser scanning confocal microscope (Zeiss). Z-series were taken with a spacing of 0.39 μm using α Plan-Apochromat 100 ×/1.46 Oil DIC M27 objective with the pinhole set to one Airy unit. A human IGS or a HAC was scored as being co-localized with the nucleoli if the green signal of a FISH probe overlapped the red signal of the nucleolar RRP1 immunostaining.

The images for the analysis were obtained as Z-stacks, projected, and analyzed visually using Fiji software. The threshold for scoring co-localization with nucleoli was the overlap or touch of two signals (as in Ref. [32]).

### Construction of lentiviral vectors

To re-activate transcription of the silent rDNA gene clusters in mouse monochromosomal hybrid cell lines, the lentiviral vectors coding transcription factors TAF1A, TAF1B, TAF1C, and TAF1D were constructed. Full-size cDNAs of the corresponding genes were amplified by PCR using a set of corresponding primers (Table S1) from plasmids kindly provided by Dr. Nagata [41]. Following digestion with AscI/SpeI enzymes, cDNAs were ligated with the AscI/SpeI treated pLVTHM-Klf4 backbone [42]. To pack the lentiviral particles, 293T cells cultivated on a 10-cm dish were transfected with 4 mg of envelope-encoding pMD2G, 8 mg of packaging pCMV-dR8.74PAX2, and 11 mg of either TAF1A-, TAF1B-, TAF1C-, TAF1D- or EGFP-encoding pLVTHM-based plasmid by the calcium-phosphate method. Lentiviruses in the cell culture supernatant were collected, processed, and tested as described elsewhere [43].

### RT-PCR analysis of gene expression from lentiviral vectors

To confirm the expression of the cDNA in the lentiviral vectors, RT-PCR was performed. First, total RNA from the infected A9 (#13) 89-2 cells was isolated using the Qiagen RNeasy plus mini kit (Qiagen, cat. no. 74034). 1 mg of purified RNA was used to synthetize cDNA using High-Capacity cDNA Reverse Transcription Kit (Thermo Fisher Scientific, cat. no. 4368813). Synthetized cDNA was checked by PCR using corresponding primers listed in Table S1.

### Lentiviral infection of A9 (#13) 89-2 cells

A9 (#13) 89-2 cells carrying the human chromosome 13 were seeded at 1.5 × 10^5^ cells per well of a 6-well plate. 0.5 ml of the viral supernatant containing the particles expressing LVTHM-TAF1A, TAF1B, TAF1C, and TAF1D were added to the cells. Two controls were done, one with untreated A9#13 89-2 human/mouse hybrid cells and another with A9#13 89-2 cells infected with LVTHM-EGFP lentiviruses. To achieve 100% efficiency, two rounds of infection were performed 48 h apart. After infection, the cells were incubated for one week before transferring to a cover glass and examination. Each lentiviral transduction was conveniently performed in one well of a 6-well plate (equal to a 3.5 cm dish). Three independent experiments were done.

### RT-PCR of long non-coding RNA NR_0038958

To check processed NR_0038958 expression, total RNA from HT1080 and A9 (#13) 89-2 cells was isolated using the Qiagen RNeasy Plus Mini Kit (Qiagen, cat. no. 74034). 1 mg of purified RNA was used to synthetize cDNA using the High-Capacity cDNA Reverse Transcription Kit (Thermo Fisher Scientific, cat. no. 4368813). RT-PCR to detect the NR_038958 transcript was carried out with primer sequences from within the IGS and DJ regions (Table S1). The primers were designed to include junctions between exon 2 and exon 3; and exon 5 and exon 6 to avoid false-positive results from genomic DNA contamination.

### Construction of TAR vectors and TAR BAC constructs

TAR cloning experiments were carried out as previously described [44]. A scheme of TAR cloning is presented in Fig. S1 (Step 1). For construction of TAR vectors, we used the basic vector pVC604 containing the yeast HIS3 gene allowing the selection of yeast transformants and a CEN sequence (CEN6) for proper propagation in yeast cells [44]. To construct TAR1, TAR2 and TAR3 vectors, the 5′ and 3′ targeting sequences (hooks) were inserted into the polylinker of the pVC604 vector as BamH1-SalI and SalI-XhoI fragments. Before TAR cloning, these TAR vectors were linearized with SalI digestion to exposure 5′ and 3′ targeting sequences. To construct TAR4 and TAR5 vectors, we performed a PCR amplification reaction with the following PCR conditions, i.e., 94 °C 1 min, 1 cycle; 94 °C 30 s, 55 °C 30 s, 68 °C 3 min, 30 cycles; 68 °C 3 min, and the corresponding primer pairs (Table S1) (Fig. S2). 42FseI-604F/42-604R primers contain hook4 and hook5 and 337PacI-604R/337BsiWI-604F primers contain hook6 and hook7. Sequences of all the primers used for TAR4 and TAR5 vectors construction are listed in Table S1.

The TAR1 vector contains a 5′ 132 bp hook1 (positions 94,985–95,116 in RP11-337M7 BAC) and a 3′ 133 bp hook3 (positions 148,974–149,106 in RP11-337M7 BAC). TAR1 vector was used to isolate a 54,121 bp fragment (positions 94,985–149,106 in RP11-337M7 BAC; GenBank AL592188.60) containing the 43,754 bp fragment of the complete copy of the rDNA unit and a 2823 bp sequence corresponding to the pre-promoter sequence involved in the regulation of rRNA transcription (positions 102,540–105,423 in RP11-337M7 BAC) (TAR1 BAC construct; Fig. S3a, b).

The TAR2 vector contains 5′ hook1 and a 3′ 101 bp hook2 (positions 120,506–120,606 in RP11-337M7). TAR2 vector was used to isolate a 25,621 bp fragment (positions 94,985–120,606 in RP11-337M7 BAC) containing a ~ 15,265 bp of the transcribed part of the rDNA unit (45S) plus a 2823 bp sequence corresponding to the pre-promoter sequence (TAR2 construct; Fig. S3a, b).

The TAR3 vector contains 5′ hook2 and 3′ hook3. The hook sequences were PCR-amplified from BAC RP11-337M7 using hook-specific primers (see Table S1). TAR3 vector was used to isolate a 28,601 bp fragment (positions 120,506–149,106 in RP11-337M7 BAC) corresponding to the IGS sequence (TAR3 construct; Fig. S3a, b).

The TAR4 vector contains a 5′ 50 bp hook4 (positions 1–50 in JH42 BAC) and a 3′ 50 bp hook5 (positions 58,328–58,377 in JH42 BAC). TAR4 vector was used to isolate a 58,377 bp fragment (positions 1–58,377 in JH42 BAC; GenBank MT497460.1) of the PJ sequence (TAR4 construct; Fig. S3a, b).

The TAR5 vector contains a 5′ 60 bp hook6 (positions 58,376–58,435 in RP11-337M7 BAC) and a 3′ 60 bp hook7 (positions 101,255–101,314 in RP11-337M7 BAC)*.* TAR5 vector was used to isolate a 42,938 bp fragment (positions 58,376–101,314 in RP11-337M7 BAC) of the DJ sequence (TAR5 construct; Fig. S3a, b).

The BAC construct IGS-Δ was obtained by ligation of the PmeI/I-SceI digested TAR4 construct (Fig. S4) and contains an 18,971 bp fragment (positions 120,506–139,477 in RP11-337M7 BAC) corresponding to the IGS sequence deleted by 9630 bp at the 3′ end of IGS. This deleted part includes pre-promoter sequences involved in the regulation of rRNA transcription.

TAR/YACs were converted into a BAC form using the shuttle vector pJBRV1 [45] and then electroporated into DH10B competent cells (GIBCO/BRL) using a Bio-Rad Gene Pulser as described [33]. BAC DNAs were purified using Plasmid Maxi Kit (Qiagen, cat. no. 12162) (Fig. S1; Step 2–Step 4).

### Physical characterization of TAR BAC constructs

Several approaches were taken to check the integrity of the TAR BAC constructs containing different inserts. First, BAC DNAs were checked by PCR using pairs of primers diagnostic for a desired region (Fig. S3c). Primers for the rDNA unit (18S, 5.8S, 28S, IGS) and detection primers for DJ and PJ regions are listed in Table S1. Second, to determine the size of the inserts in the region-positive BAC clones, BAC DNAs were digested with endonucleases that cut only in the vector portion, separated by CHEF, and stained with EtBr (Fig. S3d).

### Loading of TAR BAC constructs into the loxP site of alphoid^tetO^-HAC in hamster CHO cells

Loading of the TAR BAC constructs into the unique loxP site of the alphoid^tetO^-HAC vector propagated in the CHO1-38-18 clone [45] is described in Fig. S5a. Loading was performed using Cre/loxP-mediated recombination as described [46]. Briefly, 2 µg of an appropriate construct and a Cre-recombinase expression vector were co-transfected into Hprt-deficient hamster CHO cells (2.5 × 10^5^) carrying the alphoid^tetO^-HAC vector by lipofection using Viafect (Promega) [4.5 μl of Lipofectamine Reagent (Invitrogen Corporation)]. HPRT-positive colonies were selected after 3 weeks of growth in the HAT medium.

Insertion of TAR BAC constructs was confirmed by PCR using specific primers to detect reconstitution of the *HPRT* gene (Fig. S5b and Table S1). Integrity of the inserted material was confirmed by PCR amplification reaction using diagnostic pairs of primers corresponding to each region of interest (Fig. S5c–e and Table S1).

To confirm that the HACs are maintained autonomously in hamster CHO cells, FISH analysis was performed. The images of the HAC carrying different TAR BAC constructs in CHO cells are shown in Fig. S6. The HAC signal colocalizes with the PNA (peptide nucleic acid) FITC-labeled probe for the alphoid^tetO^ array signal on metaphase chromosome spreads. One HAC clone containing each TAR BAC construct was chosen for further analysis.

### Microcell-mediated chromosome transfer (MMCT)

MMCT transfer of the HACs carrying different TAR BAC constructs from hamster CHO cells to human HT1080 cells was performed as described in detail previously [47, 48]. Blasticidin S selection (4 μg/ml) was applied and HT1080 Blasticidin S resistant clones were picked after 2–3 weeks of growth under selective conditions. Typically, 3–7 BS^R^ colonies were obtained in one MMCT experiment involving HAC transfer from hamster to human cells. To confirm that alphoid^tetO^-HACs carrying different TAR BAC constructs propagate autonomously in human HT1080 cells without detectable integration into chromosomes, FISH analysis was performed (Fig. S6).

### Chromatin modification of HAC kinetochore by transiently expressed tetR-tTA^VP64^

1.5 × 10^5^ of HT1080 cells bearing the alphoid^tetO^-HAC (either HAC/GFP or HAC/rDNA or HAC/DJ) were seeded onto each well of a 6-well plate in 2 ml of growth medium supplemented with 5 μg/ml of Blasticidine S and 1 μg/ml of Doxycycline. Next day, the cells were transfected with 1 μg of tetR-tTA^VP64^ vector with ViaFect™ Transfection Reagent (Promega) according to the manufacturer’s protocol. Next day, the medium was changed to 10 ml of a fresh medium either with 1 μg/ml of doxycycline or without doxycycline. After 7 days of cultivation, the cells were washed with PBS, trypsinized and seated on the gelatin-covered microscopic slides. Next day, the slides were fixed and stained with the PNA FITC-labeled FISH probe for the tetO sequence in the alphoid^tetO^ array of the HAC and anti-RRP1 antibodies (Nop52) to visualize nucleoli.

### Chromatin immunoprecipitation assay (ChIP) and real-time PCR

ChIP using antibodies against trimethyl H3K4me3 (Millipore) was carried out according to a previously described method [49]. Briefly, cultured cells were cross-linked in 1% formaldehyde for 10 min at 37 °C. After the addition of 1/10 volume of 1.25 M glycine and incubation for 5 min, fixed cells were washed twice with cold PBS buffer. Soluble chromatin was prepared by sonication (Bioruptor sonicator; Cosmo Bio) to an average DNA size of 500 bp in sonication buffer and immunoprecipitated in IP buffer (20 mM Tris–HCl, pH 8.0, 600 mM NaCl, 1 mM EDTA, 0.05% SDS, 1.0% Triton X-100, 20% glycerol, 1.5 μM aprotinin, 10 μM leupeptin, 1 mM DTT and 40 μM MG132). Protein G sepharose (Amersham, USA) blocked with BSA was added and the antibody–chromatin complex recovered by centrifugation. The recovery ratio of the immunoprecipitated DNA relative to input DNA was measured by real-time PCR using a CFX96 real-time PCR detection system (Bio-Rad) and iQ SYBR Green Supermix (Bio-Rad). Primers for alphoid^tetO^ repeat (tetO) as well as for a control 5S ribosomal DNA, are listed in Supplementary Table S1. At least three independent ChIP experiments were performed to estimate the level of enrichment.

### Measuring the size of nuclear and nucleolar areas

The images obtained from 3D immuno-FISH with an LSM780 laser scanning confocal microscope (Zeiss) were analyzed using FiJi software. A polygon selection tool was used to measure nuclear and nucleolar areas in human/mouse monochromosomal hybrid cell lines A9 (#13) 89-2 (chromosome 13) and A9hygro14 10F (chromosome 14). The ratios of areas of nucleolus/nucleus were calculated using Excel software.

### Nucleofection

Nucleofection on Lonza 4D nucleofector was performed according to the manufacturer’s protocol. Briefly, SF Cell Line 4D-Nucleofector™ X Kit L (Lonza) was used. 1 × 10^6^ HT1080 cells were used per nucleofection. The primers used for the experiment are listed in Table S1.

### Statistical analysis

The statistical significance of comparisons between data groups was determined using a two-tailed nonparametric Mann–Whitney *U* test. *p* values of less than 0.05 were considered as statistically significant. Data are presented in diagrams as the mean ± SD; error bars correspond to a 95% confidence interval. *p*-values were generated for the difference in the percentage of nucleolar association. Average percentages were generated from three independent repetitions of each experiment on replicate cultures. GraphPad Prism 9 software was used for statistical analysis.

### Comparative analyses of the proximal 100 kb end of human DJ sequence

We used published GenBank annotations (http://www.ncbi.nlm.nih.gov/) of AL592188 (NT_167214) sequences that contain the proximal 100 kb end of the DJ region. Annotations from the Ensembl database (https://www.ensembl.org/) for a regulatory build as well as information from “Conservation Track” of the UCSC database (http://genome.ucsc.edu) were also used in the analysis of AL592188 (GL000220) sequences to locate orthologous hits and in primates and look for conservative blocks and regulatory sites or potential functional signals.

Multiple alignments of 30 mammalian and 100 vertebrate species were retrieved for each analyzed region of interest with a 100-nt extension on both sides from the UCSC Genome Browser (http://genome.ucsc.edu). The conservation scores of each hit or analyzed region were obtained by averaging *PhyloP* and *PhastCons* scores (“Conservation Track”), which measures conservation such that only “runs” of conserved sites are considered through the use of the hidden Markov model. BLAST (https://blast.ncbi.nlm.nih.gov/) and BLAT (http://genome.ucsc.edu/cgi-bin/hgBlat) were used to confirm the synteny of the aligned full-length sequences of the isolated human and primate blocks. Multiple alignments of nucleotide sequences were constructed using the program Muscle with default parameters [50] and edited to take into account the results of pairwise comparisons, which was done using the program OWEN [51]. Pairwise comparisons were also used to estimate the evolutionary rate for different functional regions or blocks of the DJ sequences. Evolutionary divergence was assessed by PAML [52] in pairwise alignments for each region analyzed. Comparative pairwise analysis of predicted promoter regions and exons of human LncRNA was performed with chimpanzee orthologous sequences and were also compared with other primate orthologs if their sequences were available. In all cases, our alignments contained putative transcripts reported in GenBank and UCSC. We masked the sequences using Repeat Masker (http://humangen.med.ub.es/tools/RepeatMasker.html) because the numerous low-complexity and repetitive regions in the long DJ sequences obscured the pattern of orthology. All predicted primate transcripts were analyzed using GENSCAN (http://hollywood.mit.edu/GENSCAN.html) and NCBI Orf Finder (https://www.ncbi.nlm.gov/orffinder/).

Modified DME-X (http://rulai.cshl.edu/dme) and our implementation motif search approach [53–55] were used for an extensive search of regulatory sites and signals to recognize unknown conserved blocks or sites. We also searched for regulatory sites and signals using the TRANSFAC database (https://genexplain.com/transfac/). The 5′ ends of the predicted transcripts were computationally folded using programs Afold [56] and Alifold [57] to predict potential stable structures conserved in primates. The predicted minimum secondary structure free energy was calculated using our implementation of the dynamic programming algorithm (Afold) described by Zuker, which employs nearest neighbor parameters (http://www.unafold.org/mfold/applications/rna-folding-form.php) to estimate free energy.

## Results

### The number of copies of transcriptionally silent human ribosomal genes does not affect the association of acrocentric chromosomes with nucleoli in a mouse background

To assess whether the number of rDNA repeats affects the association of acrocentric chromosomes with nucleoli, we exploited a panel of monochromosomal somatic cell hybrids in which individual human acrocentric chromosomes have been transferred into and then maintained in mouse A9-cells. The cell lines include A9 (#13) 89-2 (chromosome 13) [58]; A9hygro14 10F (chromosome 14) [58]; A9 (Neo15)-3 (chromosome 15) [58]; A9#21-16 (chromosome 21) [59]; A9#22 (γ2) (chromosome 22) [60]; and A9HyTK-22 (chromosome 22) [35].

We first demonstrated that human ribosomal genes in these hybrid cell lines are transcriptionally silent (Fig. S7). Next, we estimated the size of rDNA clusters by Southern blot hybridization analysis (Fig. 1b and Fig. S8). For this purpose, genomic DNA from each hybrid cell line was isolated and digested by EcoRV endonuclease. This nuclease has no or very rare recognition sites in human rDNA repeats, but such sites are present in the junction sequences [61]. EcoRV-digested genomic DNA was separated by CHEF and hybridized with a probe specific to the human rDNA spacer sequence (see “Materials and methods” for detail). Based on Southern blot analysis, as expected, the acrocentric chromosomes contain rDNA clusters of different size. Chromosome 13 contains three electrophoretic bands ~ 1.5 Mb, 650 kb and 250 kb in size. Chromosomes 14, 15 and 21 contain ~ 1.1 Mb, 650 kb and > 2.5 Mb bands, respectively. Chromosome 22 in cell line A9#22 (γ2) yields two bands of ~ 250 kb and 140 kb, while from the A9HyTK-22 cell line just one band of ~ 125 kb in size, suggesting content of at least two units of rDNA.

We next independently assessed the number of copies of human rDNA in each cell line by quantitative real-time PCR (qPCR) analysis, using primers specific for the human rDNA spacer sequence (Table S1). Based on qPCR, the results were consistent with the analysis of restriction digests: A9#22 (γ2) and A9HyTK-22 cell lines carrying chromosome 22 were inferred to contain 2 (95% CI 2) and 9 (95% CI 7–11) human rDNA repeats, respectively, and A9 (#13) 89-2, A9hygro14 10F, A9 (Neo15)-3, and A9 #21-16 cell lines carrying chromosomes 13, 14, 15 and 21 contained 78 (95% CI 48–108), 39 (95% CI 26–53), 17 (95% CI 14–19) and 43 (95% CI 29–57) repeats, respectively (Fig. 1d; Table S2).

The subnuclear localization status of all NORs during interphase was then examined using immunofluorescence and in situ hybridization on three-dimensional preserved nuclei (3D immuno-FISH) (see “Materials and methods”) (Fig. 1e). Nucleoli were immunostained with an anti-RRP1 antibody (Nop52).

We found that nucleolar association of acrocentric p-arms can occur in mouse background, and this association occurred independently of the amount of rDNA present on the acrocentric chromosomes. For example, the percentage of cells containing an associated nucleolar localization of chromosomes 13 and 21, which contain the highest copy numbers of the rDNA repeat (78 and 43, respectively), was 42% (95% CI 37–47%) and 48% (95% CI 42–54%), respectively (Fig. 1f and Table S3). This was almost identical to the 40% value for chromosome 22 [A9#22 (γ2) cell line] (95% CI 33–47%), which contains only nine copies of the rDNA repeat (Fig. 1f). In contrast, the percent of nucleolar localization of chromosome 22 in A9HyTK-22 cells, with two copies of the rDNA repeat, was rather lower, 30% (95% CI 20–38%). Notably, the highest percentage of nucleolar association was observed for chromosomes 14 and 15, i.e., 86% and 76%, respectively (95% CI 81–91% and 68–84%) (Fig. 1f), despite the fact that they do not have the highest copy number of the rDNA repeats (39 and 17, respectively).

As a control experiment to evaluate random associations of human chromosomes with mouse nucleoli, we tested the extent of nucleolar co-localization of non-acrocentric chromosomes 4 and 18 propagated in human/mouse hybrid cell lines A9KM10-4 and A9 48-4, respectively (Fig. 1f and Table S3). The percentage of nucleolar association observed for chromosomes 4 and 18 was 21% and 24%, respectively (95% CI 17–25% and 19–29%). The relatively high background in the experiment can be partially due to the presence of large blocks of centromeric and pericentromeric heterochromatin [62–71]. Additional factor(s), as yet unknown, must account for the rest of the background hybridization; but as we report, the hybridization attributable to portions of the rDNA repeat unit is significantly higher than the background level.

To summarize, we conclude that in the hybrid cell lines used here, NOR regions of human acrocentric chromosomes associate with mouse nucleoli but is likely not affected by the number of rDNA repeats.

### Transcription of rDNA is important for the association of NORs with nucleoli

We next asked whether re-activating transcription of the human rDNA in a murine cell background would affect the association of a NOR with nucleoli. For these experiments, we focused on the A9 (#13) 89-2 hybrid cell line containing human chromosome 13, which has the highest number of rDNA repeat (78 repeats) but a relatively low percentage of nucleolar localization in the hybrid cell nucleoli (42%).

**Fig. 2:**
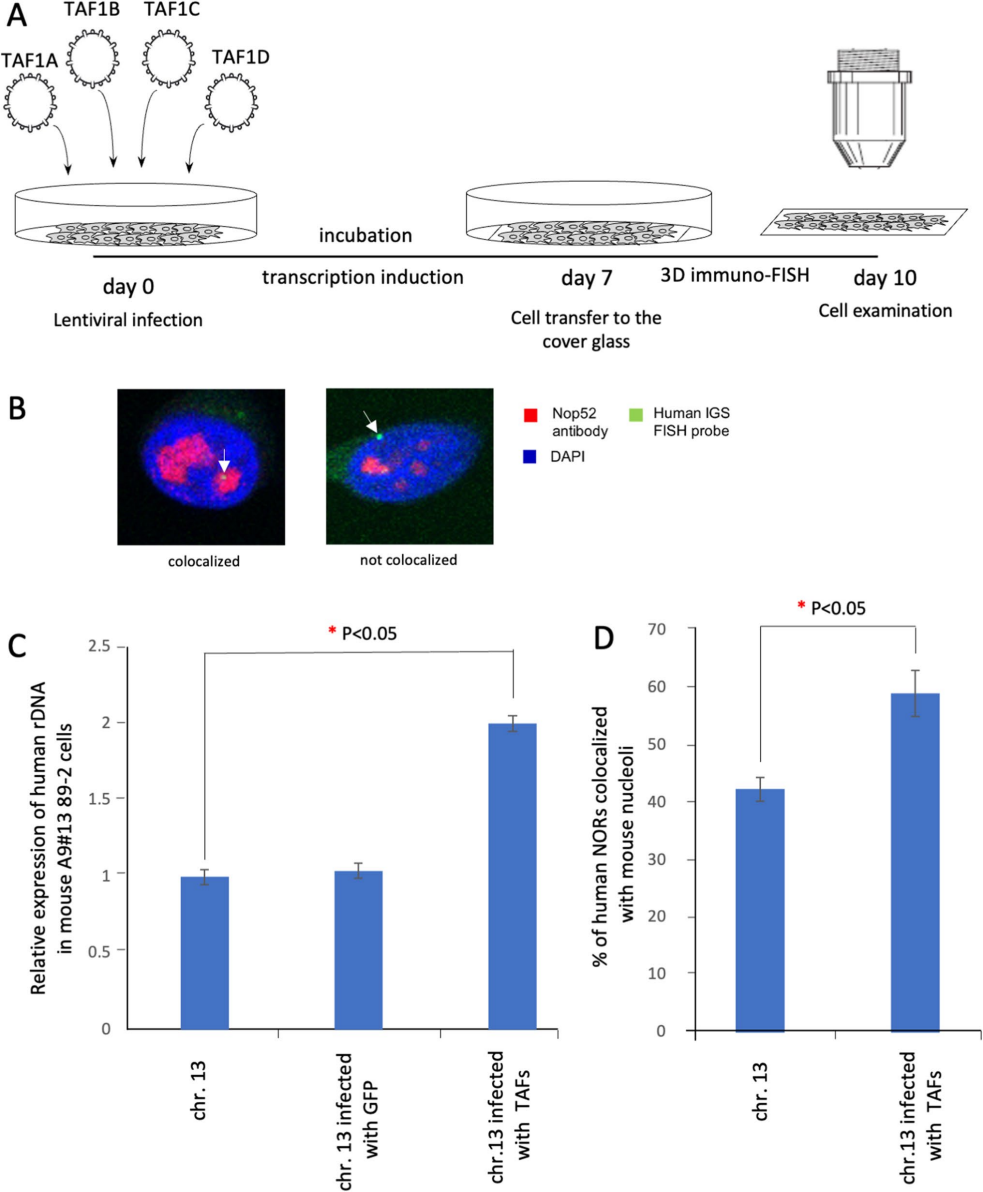
Transcription reactivation of the silent rDNA cluster from the human chromosome 13 in a mouse background. **a** Scheme of the experiment. On day 0, A9#13 89-2 human/mouse hybrid cells carrying the human chromosome 13 were infected with four lentiviruses coding transcription factors TAF1A, TAF1B, TAF1C, and TAF1D with an efficiency close to 100%. The cells were incubated for 7 days and then transferred to the gelatin-covered microscopy glass slide. After that, the 3D immuno-FISH analysis was performed. **b** 3D immuno-FISH was performed on A9#13 89-2 human/mouse hybrid cells. Anti-RRP1 antibodies (Nop52) (red) used to visualize nucleoli were combined with a BAC FISH probe containing the IGS sequence (green). Nuclei were stained with DAPI (blue). Nucleolar- and non-nucleolar-associated NORs are indicated by white arrowheads. **c**, **d** Quantification of 3D immuno-FISH analysis showing the percentage of cells in which the human chromosome 13 is associated with mouse nucleoli before and after reactivation of transcription of the human ribosomal rDNA genes. Transcription is activated twofold compared to the basic level of transcription which is considered as 1 (**b**). As a control #1, untreated A9#13 89-2 human/mouse hybrid cells by were used. As a control #2, infection of A9#13 89-2 cells by LVTHM-EGFP lentiviruses were used (**b**). The total number of the nucleolar localized and non-localized chromosomes ranges from 331 to 405 (Table S3). Percentages were calculated for replicate cultures. Significant differences were calculated using a two-tailed nonparametric Mann–Whitney *U* test. Statistically significant difference is indicated with a red asterisk (**c**, **d**)

Transcription of the 45S preribosomal RNA genes is mediated by DNA-dependent RNA polymerase I (Pol I). Mouse Pol I transcription fails on human rDNA but can proceed if human TATA-binding protein (TBP)-associated factors (hTAFIs) are provided in the mouse cells [41]. The four act in the “SL1 (selectivity factor 1) complex”, which in human contains the TATA-binding protein (TBP) plus the four TBP-associated factors: TAFIA (also known as -aka-TAFI48 and TAF1A), TAFIB (aka TAFI63 and TAF1B), TAFIC (aka TAFI110C and TAF1C) [72] and TAFID (aka TAFI41 and TAF1D) [73]. We reconstituted Pol I transcription of rDNA from human chromosome 13 by transiently expressing those hTAFIs in A9 (#13) 89-2 hybrid cells (Fig. 2a; Fig. S9). Total RNA was prepared from these cells, followed by quantitative RT-PCR using primer sets for human 45S pre-rRNA (Table S1). The experiment was repeated independently three times and the percentages obtained for the replicate cultures (Table S4).

In these mouse cells, transcription of the inactive human chromosome 13 rDNA was reactivated two-fold (compared to the basic level of transcription, set as 1) following hTAFI expression (Fig. 2c), consistent with a previously published result [41]. Next, we examined the nucleolar localization status of the human chromosome 13 in interphase hybrid cells using 3D immuno-FISH (Fig. 2b) before and after reconstitution of Pol I transcription of rDNA (see “Materials and methods”). The percentage of nucleolar localization of chromosome 13 increased from 42 to 59% (95% CI 40–44%; *p* < 0.05; human rDNA is transcriptionally silent vs 95% CI 55–63%; *p* < 0.05; Pol I transcription active; 1.5-fold increase) (Fig. 2d and Table S4). We conclude that human ribosomal rRNA transcription may contribute to the association of acrocentric chromosomes with nucleoli.

We note that the extent of association of human rDNA shown in Fig. 1f ranges from 30 to 85%. This very broad range does not correlate with the sizes of the human rDNA arrays. Upon introducing human SL1 components to re-activate the transcription of human rDNA of chromosome 13 (see Fig. 2c), association goes up, reaching ~ 59%; but this value is still far below the 85% association seen with chromosome 14 in an inactive state. We considered one potential explanation, given the high degree of aneuploidy in A9 cells: nucleoli might be of different sizes in different cell lines, and in cells with larger nucleolar areas the scoring of overlaps would be higher. To address this, we measured nuclear and nucleolar areas in A9 (#13) 89-2 cells containing chromosome 13 and in A9 hygro14 10F cells containing chromosome 14. As seen from Fig. S10 and Table S5, the size of nuclear and nucleolar areas in these cell lines is the same. We thus infer that a two-fold increase of rDNA transcription in A9 (#13) 89-2 cells may be not enough to reach the level of 85% association seen with chromosome 14.

### Association of HAC with nucleoli is driven by both rDNA and non-rDNA sequences

To examine the role of DJ and PJ regions as well as rDNA sequences in the association of NORs with nucleoli, we isolated these regions by TAR cloning as circular YAC/BAC molecules [44] and then loaded them into the Human Artificial Chromosome (HAC) vector [46] (see “Materials and methods” for details).

**Fig. 3:**
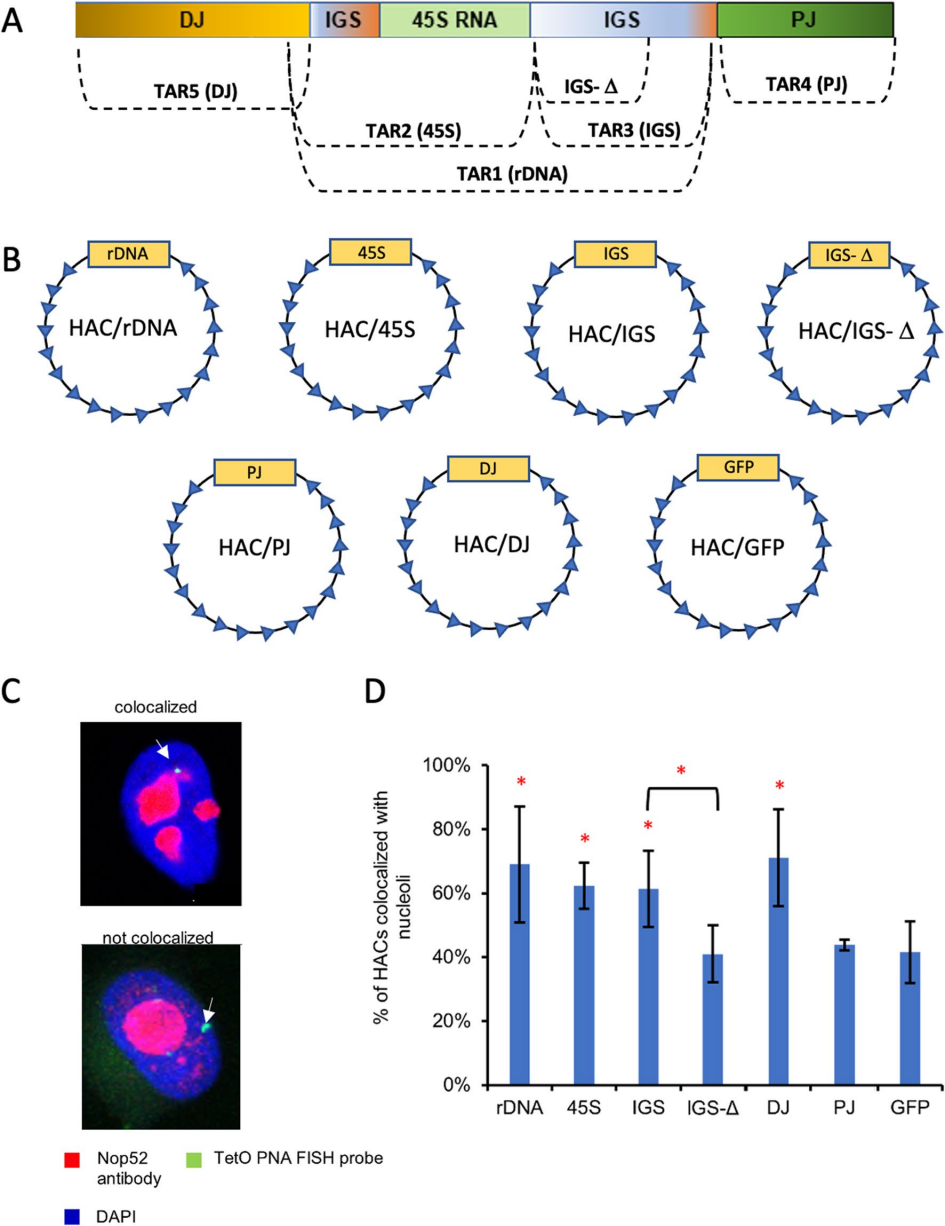
**a** TAR isolation of the rDNA repeat (rDNA; TAR1 construct), transcribed (45S; TAR2 construct) and non-transcribed (IGS; TAR3 construct) parts of the rDNA repeat, proximal junction (PJ; TAR4 construct) and distal junction (PJ; TAR5 construct) regions, and the IGS-Δ construct in which the 3′ end of the IGS sequence is deleted (see “Materials and methods” for detail). **b** Schemes of the HAC vector carrying either the rDNA repeat (HAC/rDNA), transcribed (HAC/45S) and non-transcribed (HAC/IGS) parts of the rDNA repeat, the PJ fragment (HAC/PJ), the DJ fragment (HAC/DJ) or the IGS-Δ BAC construct (HAC/IGS-Δ). A HAC carrying the *GFP* transgene (HAC/GFP) was used as a control. **c** 3D immuno-FISH in human HT1080 cells. Anti-RRP1 antibodies (Nop52) (red) used to visualize nucleoli were combined with the PNA probe for the alphoid^tetO^ array of the HAC (green). Nuclei were stained with DAPI (blue). Nucleolar- and non-nucleolar-associated NORs are indicated by white arrowheads. **d** Quantification of 3D immuno-FISH showing the percentage of cells in which the HACs carrying different constructs are associated with mouse nucleoli. For each construct, the experiment was replicated three times (Table S5). Significant differences were calculated using a two-tailed nonparametric Mann–Whitney *U* test. Statistically significant differences are marked with red asterisks

TAR constructs were selectively isolated in yeast by TAR vectors containing targeting sequences (hooks) that have homology to the 5′ and 3′ ends of a region of interest (Fig. S1 and Fig. S3). The TAR1 vector was used to isolate a 54,121 bp fragment containing the 43,754 bp sequence of a complete copy of the rDNA unit (TAR1 construct). The TAR2 vector was used to isolate a 25,621 bp fragment containing ~ 15,265 bp of the transcribed part of the rDNA unit (45S rRNA) (TAR2 construct). The TAR3 vector was used to isolate a 28,601 bp fragment corresponding to the IGS sequence (TAR3 construct). The TAR4 vector was used to isolate a 58,377 bp fragment of the PJ sequence (TAR4 construct). The TAR5 vector was used to isolate a 42,938 bp fragment of the DJ sequence (TAR5 construct) (Fig. 3a); the TAR-cloned DJ sequence is located immediately proximal to the rDNA array. Because each TAR construct has YAC and BAC cassettes, they can be propagated in yeast or in bacterial cells. Each TAR construct has a 3′ HPRT-loxP-GFP sequence that allows it to be inserted into a unique loxP site of the alphoid^tetO^-HAC in hamster donor CHO cells following the transfer of the HAC constructs into human HT1080 cells (HAC/rDNA, HAC/45S, HAC/IGS, HAC/IGS-Δ, HAC/PJ HAC/DJ and HAC/GFP) (Fig. 3b and Fig. S5). The alphoid^tetO^-HAC carrying different inserts propagates and segregates autonomously in human HT1080 cells [45].

To determine the nucleolar association status of the HACs carrying different TAR constructs within interphase cells, we applied 3D immuno-FISH (Fig. 3c) (see “Materials and methods”) (Fig. 3c). Next, we determined the percentage of the HACs that are colocalized with nucleoli during interphase (Fig. 3d). For each HAC construct, the experiment was replicated three times. The percentages were calculated for replicate cultures (Table S6). The highest percentage of nucleolar association was observed for the HACs carrying the 54 kb entire rDNA repeat (69% vs 42%; *p* < 0.05; 1.6-fold increase) and the 43 kb DJ fragment (71% vs 42%; *p* < 0.05; 1.7-fold increase) compared to 42% for the control HAC carrying the GFP transgene (Table S6). Surprisingly, the nucleolar association of HACs carrying the 26 kb transcribed (45S) and 28 kb non-transcribed (IGS) parts of the rDNA repeat was also statistically higher compared to the control (62% and 61% vs 42%, respectively; *p* < 0.05; 1.5-fold increase). In contrast, the nucleolar association of a HAC carrying the 58 kb PJ fragment did not differ from the control (44% vs 42%, respectively). In all cases, the HAC centromere is the same, and we therefore attribute the increase in association to the inserted DNA, on the assumption that any effect of centromeric heterochromatin is the same for the various constructs.

A relatively high percentage of the control alphoid^tetO^-HAC (HAC/GFP) co-localization with the nucleolus observed in these experiments is predictable. First, the HAC size is significantly smaller compared to the chromosomes that facilitates its penetration into the nucleolus. Secondly, a functional centromere in the HAC as well as in the chromosomes contains large blocks of heterochromatin. It is known that perinucleolar heterochromatin is composed of satellite DNA surrounding NORs [35]. Therefore, as expected, the HAC associates with the nucleolus.

Collectively, our analysis of HACs carrying different rDNA constructs revealed that a single rDNA repeat, its transcribed (45S) and non-transcribed (IGS) parts, and also sequences within the DJ DNA fragment can specify nucleolar association of these HACs.

What is common between 45S and IGS regions that facilitates the comparable promotion of HAC localization to nucleoli? Both transcribed and non-transcribed rDNA regions contain homologous sequences corresponding to upstream control elements and enhancers of the Pol I promoter. These sequences bind to the transcription factor UBF1, which is critical to initiate the transcription of 45S ribosomal RNA and mediates the formation of transcriptionally active chromatin in the nucleoli. To check if these sequences are important for HAC association with the nucleolus, we constructed a HAC carrying the IGS-Δ BAC construct (Fig. S4), in which the 3′ end of the IGS sequence, which includes pre-promoter sequences involved in regulating rRNA transcription, was deleted. 3D immuno-FISH in HT1080 cells revealed a significant reduction of association of this HAC with nucleoli (to the control level) (61% vs 41%, respectively; *p* < 0.0001; 1.5-fold) (Fig. 3d and Table S6). Based on these results, we infer that sequences involved in regulating ribosomal rRNA transcription are important for chromosome co-localization with the nucleoli.

### Chromatin status surrounding rDNA may be important for NORs association with nucleoli

Next, we asked whether, in addition to the effect of transcription regulation of the ribosomal DNA, the modification status of chromatin surrounding rDNA affects NOR association with nucleoli. To address this question, we exploited the alphoid^tetO^-HAC as a model in which chromatin status can be changed by specific manipulations. The alphoid^tetO^-HAC kinetochore is formed on a 1.1 Mb size synthetic alphoid^tetO^ array that contains ~ 6000 copies of the 42-bp tetracycline operator (tetO) sequence replacing the CENP-B box in each amplified synthetic dimer [39, 74]. Because tetO is bound with high affinity and specificity by the tet repressor (tetR), the HAC kinetochore can be targeted specifically with tetR-fusion proteins [39]. Binding tetR to tetO sequences can be eliminated by adding doxycycline to the medium. Thus, the synthetic alphoid^tetO^-HAC allows the targeted manipulation of chromatin composition within a single functional centromere in vivo without affecting all kinetochores of the endogenous chromosomes of the host cell.

**Fig. 4:**
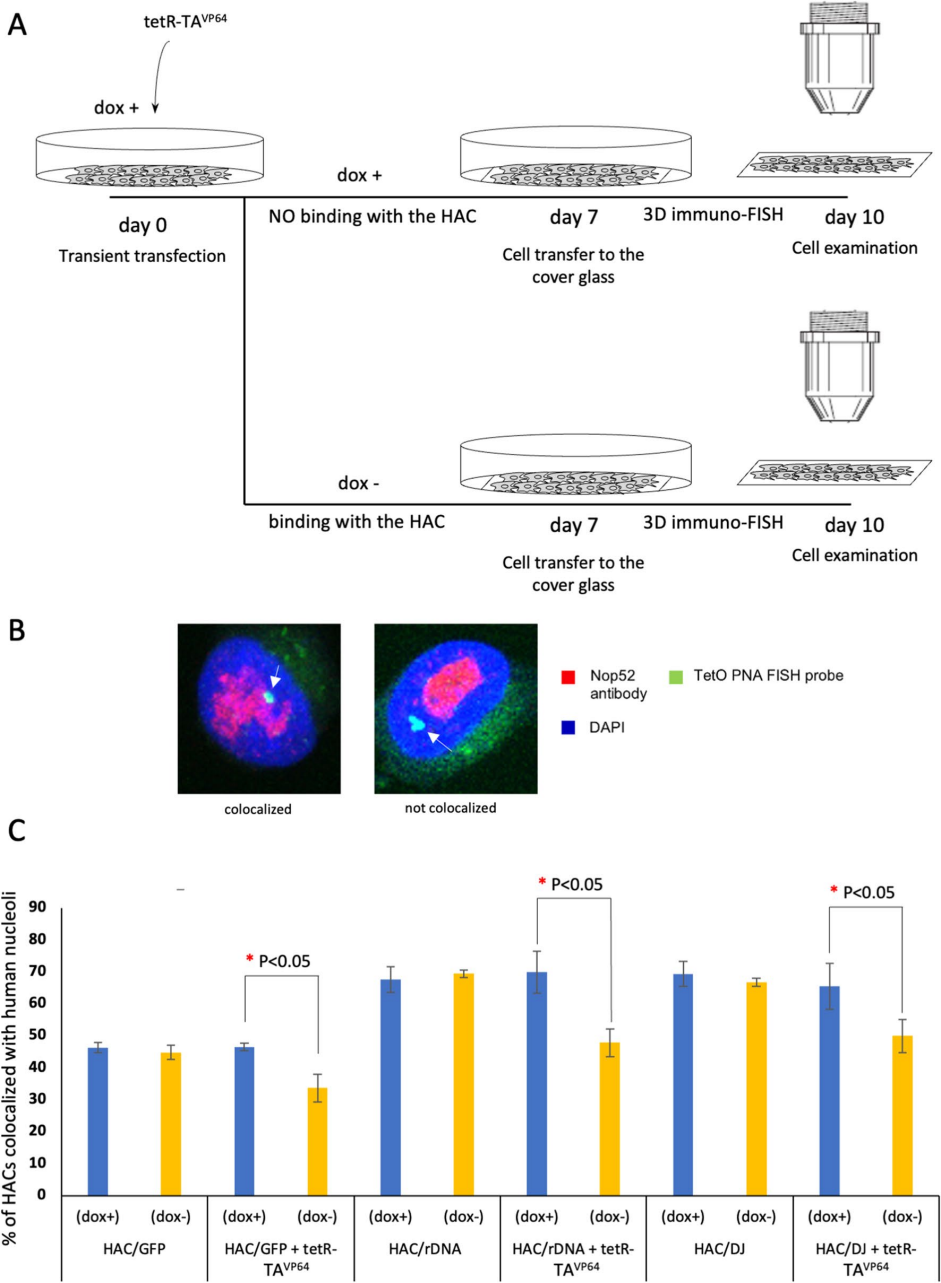
Effect of chromatin status on HAC localization in the nucleolus in human HT1080 cells. **a** Scheme of the experiment. On day 0, the cells with HAC/GFP, HAC/rDNA and HAC/DJ were transfected with tetR-tTA^VP64^ with an efficiency close to 100%. Cells were incubated for 7 days in the presence or absence of doxycycline and then transferred onto the gelatin-covered microscopy glass slide. On day 10, 3D immuno-FISH was performed. **b** 3D immuno-FISH with anti-RRP1 antibodies (Nop52) (red) and the PNA probe for the alphoid^tetO^ array of the HAC (green). Nuclei were stained with DAPI (blue). Nucleolar- and non-nucleolar-associated HACs are indicated by white arrowheads. **c** Relative changes of HACs colocalization with the nucleoli in dox-plus versus dox-minus medium after transfection by the tetR-tTA^VP64^ fusion protein genes. Transfection by tetR-tTA^VP64^ in a dox-minus medium leads to its binding to tetO sequences on the HAC kinetochore and reduction of HAC/rDNA and HAC/DJ association with the nucleoli (1.5-fold and 1.5-fold decrease, respectively). Significant differences were calculated using a two-tailed nonparametric Mann–Whitney *U* test. Statistically significant differences are indicated with red asterisks

In this study, we used transient transfection of tetR-TA^VP64^, i.e., tetR fused to the advanced *tTA*^*VP64*^ (tet-repressor transcriptional activator containing four tandem repeats of the VP16 domain), that can generate chromatin changes in the HAC kinetochore due to elevation of transcription of the alphoid^tetO^ array which rapidly inactivates the HAC kinetochore [39, 49]. In our experiments, we used the alphoid^tetO^-HAC carrying the 54 kb entire rDNA repeat (HAC/rDNA), the HAC carrying the 43 kb DJ sequence (HAC/DJ) and the HAC carrying the GFP transgene (HAC/GFP). The HACs are propagated in human HT1080 cells. To change the chromatin status of the entire 1.1 Mb alphoid^tetO^ array in the HAC/rDNA, HAC/DJ and HAC/GFP, we transiently transfected the multiple copy vector expressing tetR-tTA^VP64^ into HT1080 cells bearing the HACs in the non-selective medium either with doxycycline (dox^+^) to inhibit binding of tetR-tTA^VP64^ to the alphoid^tetO^ array sequence of the HACs or without doxycycline (dox^−^) to permit tetR-tTA^VP64^ binding (Fig. 4a). After that, the sub-nucleolar localization of the HACs was determined using 3D immuno-FISH (Fig. 4b) (see “Materials and methods”).

First, we carried out a control experiment to show any change in the chromatin status of the alphoid^tetO^-array, which forms a functional centromere on the HAC, after transient transfection of tetR-TA^VP64^ in dox− medium. ChIP with antibodies against trimethyl H3K4me3 using the primers for alphoid^tetO^ repeat (tetO) and as a control locus, for *5S* ribosomal DNA (Table S1) revealed elevation of satellite transcription of the alphoid^tetO^ array accompanied by enrichment in H3K4me3 chromatin (Fig. S11), in agreement with previously published data [49]. Three independent ChIP experiments were performed to estimate the level of enrichment.

Next, for each HAC construct, association with nucleoli was determined with and without tetR-tTA^VP64^ transfection. As expected, without tetR-TA^VP64^ transfection no significant difference in HAC association with nucleoli was observed when cells were cultured in dox+ and dox− media (46% vs 44% for HAC/GFP, 68% vs 69% for HAC/rDNA and 69% vs 67% for HAC/DJ) (Fig. 4c and Table S7). Transient transfection of cells with a vector expressing tetR-TA^VP64^ had essentially no effect on HAC/GFP, HAC/rDNA and HAC/DJ association with the nucleoli in the presence of doxycycline (when tetR-TA^VP64^ binds to doxycycline and becomes non active) (Fig. 4c). In contrary, tetR-TA^VP64^ expression in doxycycline-minus media caused a statistically significant decrease of HAC/GFP association with the nucleoli (46% vs 34%; *p* < 0.05; 1.4-fold decrease) (Fig. 4c and Table S7). Comparable effects were observed for the HAC carrying either the rDNA unit or the DJ sequence (70% vs 48%; *p* < 0.05; 1.45-fold decrease and 65% vs 49%; *p* < 0.05; 1.3-fold decrease, respectively) (Fig. 4c and Table S7). Thus, we infer that heterochromatin alterations in the HAC kinetochore affect the localization of the HAC molecules in nucleoli.

Finally, we determined sub-nucleolar localization of the HAC/GFP and HAC/rDNA before and after altering their chromatin state to a more open configuration with the tTA^VP64^ transcriptional activator. 3D immuno-FISH revealed that in both cases the HACs are located preferentially in perinucleolar heterochromatin (80–84%) (Table S8)*.* Thus, chromatin changes in the HAC centromere induced by the function of a transcriptional activator do not alter peripheral localization of the HAC in the nucleolus.

### Comparative analysis of the DJ sequences located immediately proximal to rDNA

As shown above, sequences within a 43 kb DJ DNA fragment located immediately proximal to the rDNA array specify nucleolar association of the HAC. Therefore, we decided to carefully analyze the proximal 100 kb of the DJ sequence in vertebrate genomes and also consider whether this region is conserved in evolution.

**Fig. 5:**
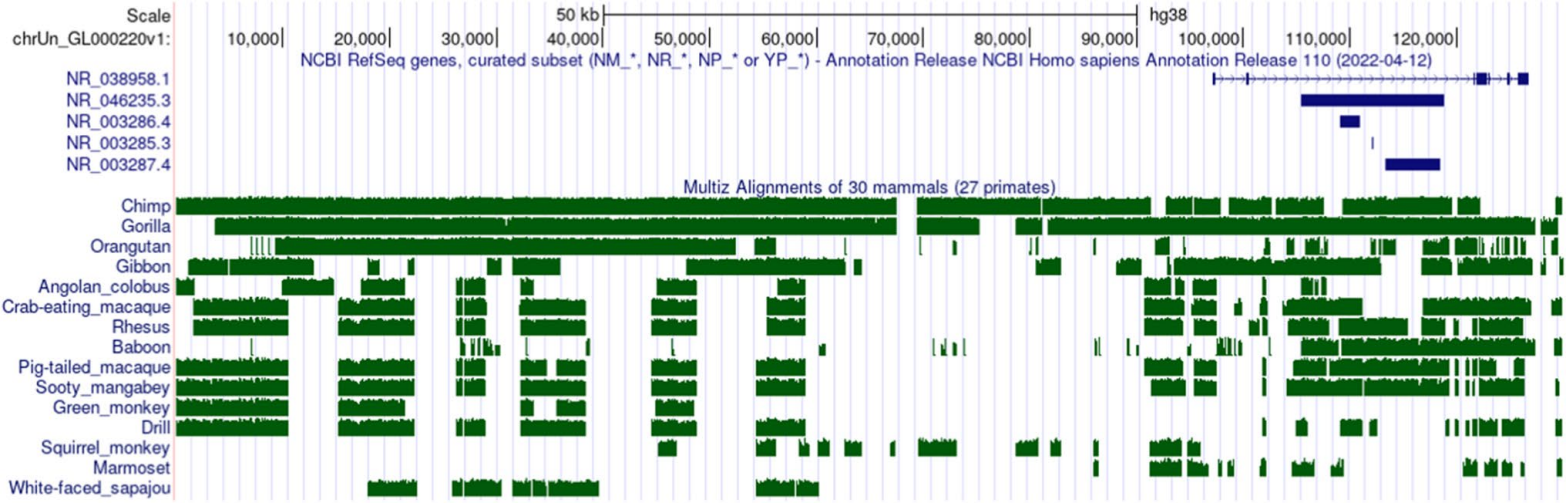
Comparative analysis of the proximal 100 kb sequence of the human DJ region with primate genomes. Schematic representation of the proximal end of the human DJ region (contigs IDs: GL000220 and AL592188.60) and its comparative analysis are based on UCSC genomic data (https://genome.ucsc.edu/). The top 15 primate genomes with unambiguous levels of similarity/identity are presented, based on multiple alignments of 30 mammalian genomes. The 15 pairwise alignments of each specie with the human genome (green boxes) are shown below the NCBI RefSeq annotation of the genes as a wiggle track (“Multiz Alignments of 30 mammals”), where height indicates an alignment quality. NCBI annotations of seven exons of LncRNA NR_038958 located in the proximal region of DJ and other noncoding RNAs, including ribosomal RNAs, are shown as blue boxes

The DJ sequences were previously shown to be predominantly confined to acrocentric short arms and are dominated by a large, inverted repeat of > 100 kb, in which a large (~ 40 kb) block of 48-bp satellite repeats, CERs, was identified at the distal end of the DJ sequence [32]. Here we analyzed the proximal end of the DJ region and showed that in addition to the CER blocks, which occur distal to the rDNA on all acrocentric chromosomes, additional conserved elements and blocks are found in the proximal 100 kb of the human DJ region. Multiple and pairwise alignments of the human and primate DJ regions from the proximal end were created based on the UCSC comparative genomic data for 30 mammals and 100 vertebrates’ comparisons (Fig. 5 and Fig. S12a). Alignment revealed that the first 100 kb of DJ contigs contain highly conserved blocks that form a chain with a significant similarity between human and some sequenced and annotated genomes of great apes, including chimpanzees, gorillas, and orangutans. Another group with the conserved blocks within the DJ region includes the gibbon, several species of macaques, and other Old World monkeys, including baboons, as well as some New World monkey species, such as marmosets and squirrel monkeys. Sequence alignment analysis in primates showed that numerous conserved blocks with almost identical breakpoints and indel locations are found in the region (Fig. 5).

Some conserved blocks were used for further analysis (see “Materials and methods” for details). We predicted potential open reading frames (ORFs) in the conserved regions and calculated coding potential, including PhyloP scores, for some of the most conserved potential microproteins or coding regions (fttp://ncbi.nlm.nih.gov/pub/shabalin/DJ_Region/; Table S9). We also evaluated the RNA folding stability of highly conserved regions between humans and primates with low PhyloP scores in a 100-way comparison and showed that the local stability of many conserved regions was significantly higher than that of random regions with the same nucleotide content.

LncRNA NR_038958 was described in RefSeq and Ensemble databases, based on a computational prediction that is confirmed by EST and RNA-seq data. The mature transcript is 2945 bp long and starts at the proximal end of the DJ region. It has seven predicted exons, with the first two exons located at 95,557–95,886 and 98,691–98,969 (in NW_021160026.1) in close proximity to the rDNA array, and the last five exons located in the IGS region, downstream of the rDNA transcript (Fig. S15a). The complete rRNA transcript is ~ 13 kb long and overlaps with the third intron of this LncRNA. Consensus folding for multiple alignments of the 5′ end of predicted LncRNA NR_038958 in four primate species is demonstrated in Fig. S13. These results suggest that some of the conserved blocks in the DJ region may be functional and even potentially transcribed. The sequence data described here indicate that all acrocentric chromosomes are in principle capable of producing the LncRNA NR_038958 transcript. Using reverse transcriptase PCR (RT-PCR), we experimentally confirmed the existence of the spliced transcript in HT1080 cells and its absence in the human/mouse hybrid A9#13 89-2 (Fig. S15a, b).

Motif search analysis and prediction of transcription factor (TF) sites (see “Materials and methods” for details) allowed identifying potential regulatory regions in human DJ sequences. An overall diagram of the predicted TF sites is shown in Fig. S12b with detail for the 92.5–94.5 kb region (in GL000220), which is enriched with potential TF sites and located in close proximity to the rDNA array. Experimental support has been demonstrated for some of these sites (https://www.ensembl.org/) for at least some cell lines or cultures. These sites are likely functional and may be involved in the regulation and structural determination or support of the DJ sequence architecture. One of the features that may be involved in the regulation of rDNA biogenesis and their transport in nucleoli is the CTCF sites, which are conserved and occur twice in the 92.5–94.5 kb region (Fig. S12b). The consensus C and G rich motif for the CTCF interactions was found to be highly conserved not only in humans but also in some primate genomes. A list of elements conserved between human and chimpanzee and experimentally validated TF sites in the 92.5–94.5 kb region (in GL000220) is presented in Table S10. This region is located upstream of the predicted LncRNA NR_038958 and may be involved in the regulation of transcription not only of this LncRNA but also in the regulation of expression of overlapped rRNA transcripts.

Many other potential TF site were identified near the 6–8 kb region in the GL000220 sequence, some of them were experimentally confirmed (Fig. S14a) and can also be considered as a promising area for further analysis, given the experimental support for expression in this region. Two additional conserved sequences enriched with potential sites for protein binding are located between 32 and 50 kb (shown in Fig. S14b). The most common motifs found at the proximal end of the DJ region by the DME-X program are shown in Table S11. This suggests that common motifs with high similarity to known TF sites may be responsible for protein binding and involved in the regulation of rDNA array transcription, replication, and/or transport.

To summarize, TF sites, the cluster of CTCF sites, and the LncRNA start (NR_038958) are located immediately adjacent to the rDNA array within the same 10–12 kb region. Therefore, future analysis of this region may reveal factors determining the association of the DJ and NORs with the nucleoli and possibly additional functions.

## Discussion

In this study, we have carried out a detailed analysis to clarify parameters involved in the association of the p-arms of acrocentric chromosomes with nucleoli. Earlier McStay et al. [75] assayed the qualitative content of rDNA in the p-arms of different acrocentric chromosomes in mitotic spreads of normal cells. They observed that rDNA repeats varied greatly in number between chromosomes, and saw no detectable rDNA in some NORs, suggesting that very little rDNA—likely one repeat or less—might be enough to seed nucleolar structure. In one case, a single chromosome 22 in hTert-RPE1 cells, with one DNA repeat or less, was localized with a nucleolus. From these results, they proposed that NORs localize with nucleoli even with very little—or perhaps no—rDNA content [75].

To clarify which parameters of NOR may contribute to association with nucleoli, we exploited the human artificial chromosome, alphoid^tetO^-HAC, which behaves as an extra 47th chromosome with proper segregation and replication in human cells [37]. It is important that the structure and functional domains of the alphoid^tetO^-HAC remain unchanged after HAC transfer from donor hamster CHO cells to different host mammalian cells [76]. Thus, this HAC is an ideal model to transmit and study different regions of the NOR for their potential role in nucleolus localization.

The alphoid^tetO^-HAC carrying either an entire rDNA unit, its transcribed (45S) or non-transcribed (IGS) parts, DJ or PJ DNA regions propagates in human fibrosarcoma HT1080 cells, which are very similar to non-transformed human cells in their NOR chromosomal organization and activity status [75]. Our analysis revealed that just a single rDNA repeat is sufficient to promote the nucleolar association of the HAC. Unexpectedly, 45S rDNA and IGS regions alone also promote the association of the HAC with the nucleolus. Moreover, a HAC carrying only a ~ 43 kb DJ DNA fragment is nucleolar-associated, supporting the previous inference that acrocentric p-arms associate with nucleoli independent of their rDNA content [75]. Considering all these findings, we conclude that the association of multiple acrocentric p-arms into a large mature nucleolus is a complex process that is driven by the activity and properties of both rDNA and non-rDNA sequences (the DJ).

We assume that inclusion in a nucleolus in mouse and human cells will follow the same rules. A caveat, however, is that the genomic architecture of the mouse rDNA-containing chromosomes is quite different from human acrocentric chromosomes. Normal mouse chromosomes are telocentric, with the rDNA located distal to the centromere. The surrounding satellite environment is also different from the human context. Furthermore, DJ-like sequences are features of primate genomes, while nucleoli are present in all eukaryotes. Still, mouse and human nucleolar structure and activity involve rDNA units with a similar structure ([77] and unpublished data) and many of the same core nucleolar components like fibrillarin, acidic protein C23, and RNA polymerase I; this is consistent with the possibility that sequences bracketing the rDNA repeats will also be involved in nucleolar association in mouse.

To address the question whether the heterochromatin status of NORs is important for nucleolar association of the acrocentric p-arms, we used as a model the alphoid^tetO^-HAC carrying either a full rDNA repeat (44 kb) or a short proximal DJ fragment (43 kb). As has been shown previously, the majority of the megabase-order alphoid DNA array in the alphoid^tetO^-HAC is covered by centrochromatin and heterochromatin [39, 78]. The balance between an open (for centromere) and closed (for heterochromatin) chromatin state of the alphoid DNA is important for kinetochore function [39, 79, 80]. An alphoid array of the alphoid^tetO^-HAC consisting of alpha-satellite repeats contains thousands of tet operator sequence (tetO) in every other alphoid monomer. This provides a unique feature for this HAC: its chromatin status can be changed by tethering the tetR-fusion chromatin modifiers [39, 46, 79, 80]. TetR tethering can be controlled by doxycycline in the medium. This prevents tetR from binding to tetO sequences and modulates the activity of tetR fusion proteins. In this work, we found that tethering of tetR-tTA^VP64^, which induces open chromatin thereby disrupting the HAC heterochromatin, resulted in reduction of HAC association with the nucleoli. This result suggests that NOR association with the nucleoli may be affected by the status of heterochromatin blocks formed next to the rDNA arrays.

In extending such analyses, we were interested in determining the requirements for the inclusion of rDNA repeats in nucleoli. We further capitalized on the use of a set of human/mouse hybrid cell lines, each containing one human rDNA-containing chromosome. We began by checking the rDNA content of the human acrocentric chromosomes in the hybrid cells employed in the study. Our analysis revealed a widely varying distribution of rDNA repeats between acrocentric p-arms that ranges from 2 to 78 repeats. In these hybrid cells, human ribosomal genes are transcriptionally silent, but acrocentric p-arms are associated with mouse nucleoli, which confirmed the earlier report [35]. What was new and possibly unexpected was that the degree of association does not correlate with the copy numbers of the rDNA repeat on the chromosomes. For example, human chromosome 13, which contains the largest rDNA cluster, consisting of 78 rDNA repeats, exhibited a relatively low co-localization with the mouse nucleoli. However, after re-activation of the human rDNA transcription in mouse cells by expressing human TBP-associated factors (TAFIs), the nucleolar association of chromosome 13 increased. Thus, we conclude that rather transcription but not copy number of rDNA repeats may contribute to the association of the acrocentric chromosomes with the nucleoli. This conclusion is supported by our data on the HAC containing a rDNA repeat in which the regions corresponding to the upstream control elements and enhancers of the Pol I promoter, that are involved in the regulation of transcription of the ribosomal rRNA genes, were deleted. When those regulatory sequences were deleted, we observed a significant reduction of HAC association with the nucleoli, which dropped to control levels.

As we have shown, a relatively small size DJ region (43 kb) located just next to the rDNA array is involved, along with the rDNA repeats, in the localization of p-arms of acrocentric chromosomes in nucleoli. In our study, we identified LncRNA NR_038958, a transcript starting at the proximal end of the DJ region, whose first two exons are located in close proximity to the rDNA array, and which last five exons are in the IGS region. This LncRNA is present in all acrocentric chromosomes and is located in the same region. The region between 92.5–96.5 (in GL000220) upstream of the LncRNA is enriched in TF binding sites that are conserved between human and primates and that may be involved in the regulation of transcription of this LncRNA as well as in the regulation of rRNA transcripts.

The search for DNA binding motifs revealed multiple CTCF binding sites across the 100 kb DJ region located in close proximity to the rDNA array, consistent with data previously obtained [32]. Interestingly, a cluster of CTCF binding sites is located close to the DJ/rDNA boundary and potentially promote the region of LncRNA NR_038958. The DNA-binding protein CTCF is known to be involved in many cellular processes [81], including the transcriptional regulation of ribosomal genes [82] and human nucleolar organization [83]. In cultured cells, the absence of CTCF resulted in the reduced association of UBF with rDNA and in nucleolar fusion. As mentioned above, the DJ/rDNA boundary may be an important regulatory region not only for 45S pre-ribosomal RNAs, but may also regulate the LncRNA, described and experimentally confirmed in our study (NR_038958). A detailed analysis of this region could certainly warrant further study.

Given that LncRNA NR_038958 can partially overlap the genomic location of the antisense transcripts, termed PAPAS (“promoter and pre-rRNA antisense”) found earlier in independent studies of mammalian rDNAs, we can assume a cumulative regulatory effect of these two LncRNAs in regulating the epigenetic signature at the rDNA enhancer and promoter, and an effect on RNA Pol I transcription. PAPAS is a long noncoding RNA (LncRNA) that is transcribed in an antisense orientation to pre-rRNA and produces transcripts > 10-kb in size covering the gene body and intergenic regions, including the rDNA promoter and upstream enhancer sequences. PAPAS has been shown to interact directly with DNA by forming a DNA–RNA triplex structure that bind PAPAS to a purine-rich site in the enhancer region, thereby directing the associated CHD4/NuRD (nucleosome remodeling and deacetylation) to the rDNA promoter [84–87].

Sequence identity among the DJ contigs of acrocentric chromosomes allowed us to design an antisense oligonucleotide to target the depletion of the transcript. RT-PCR analysis showed that after its transfection into HT1080 (“Materials and methods”), the transcript was indeed depleted only 50–60% (Fig. S15d) that is probably not enough because there was no drop in cell numbers compared to nontransfected cells after 96 h (Fig. S15e), and no difference in HAC association with nucleoli (Fig. S15c, f, g). Further analysis is needed to clarify if the LncRNA NR_038958 transcript has a potential role in nucleolar function and/or regulation of RNA Pol I transcription.

Despite the different ordering and identified rearrangements of DJ regions in some genomes of great apes, our comparative analysis showed ~ 96% identity between chimpanzees and humans in the first 100 kb of the DJ sequences. Another group of genomes with conserved blocks within the DJ sequences, especially in the potentially functional and important for regulation region described above, are located between ~ 92.5 kb and the beginning of 45S pre-ribosomal RNA, includes gibbon, gorilla, orangutan, and other Old World monkeys. Sequence alignment analysis in primates showed that there are numerous conserved blocks with almost identical breakpoints and indel locations in the DJ region, even though they originate from different species, including some New World monkeys. Thus, rDNA arrays and DJ sequences remain fairly conserved in primates over evolutionary time. Therefore, we assume that, like in humans, the NORs of great apes should be considered in molecular terms as being comprised of rDNA arrays and associated DJ sequences. This assumption is consistent with our finding that rDNA and DJ sequences are likely to drive nucleolar association of acrocentric p-arms in human.

The current findings provide a basis for future studies of each identified parameter and further analysis is required to understand the specific nature of interactions between all these sequences, heterochromatin, and their association with the NOR. This may further facilitate efforts to investigate how rearrangements in rDNA and DJ sequences within acrocentric p-arms or other NOR abnormalities can affect disease susceptibility.

## Supplementary Information

Below is the link to the electronic supplementary material.

Supplementary file1 (DOCX 58004 KB)

## Data Availability

All the data are available in the supplementary materials.

## Materials availability

All materials are available upon request.
