# Supplementary material for: Actively transcribed rDNA and distal junction (DJ) sequence are involved in association of NORs with nucleoli
Source: Cell Mol Life Sci. 2023 Apr 12;80(5):121. doi: 10.1007/s00018-023-04770-3 (PMC10097779; doi:10.1007/s00018-023-04770-3)
Supplement: Supplementary file 1 — Supplementary file1 (DOCX 58004 KB) [file 18_2023_4770_MOESM1_ESM.docx]

0
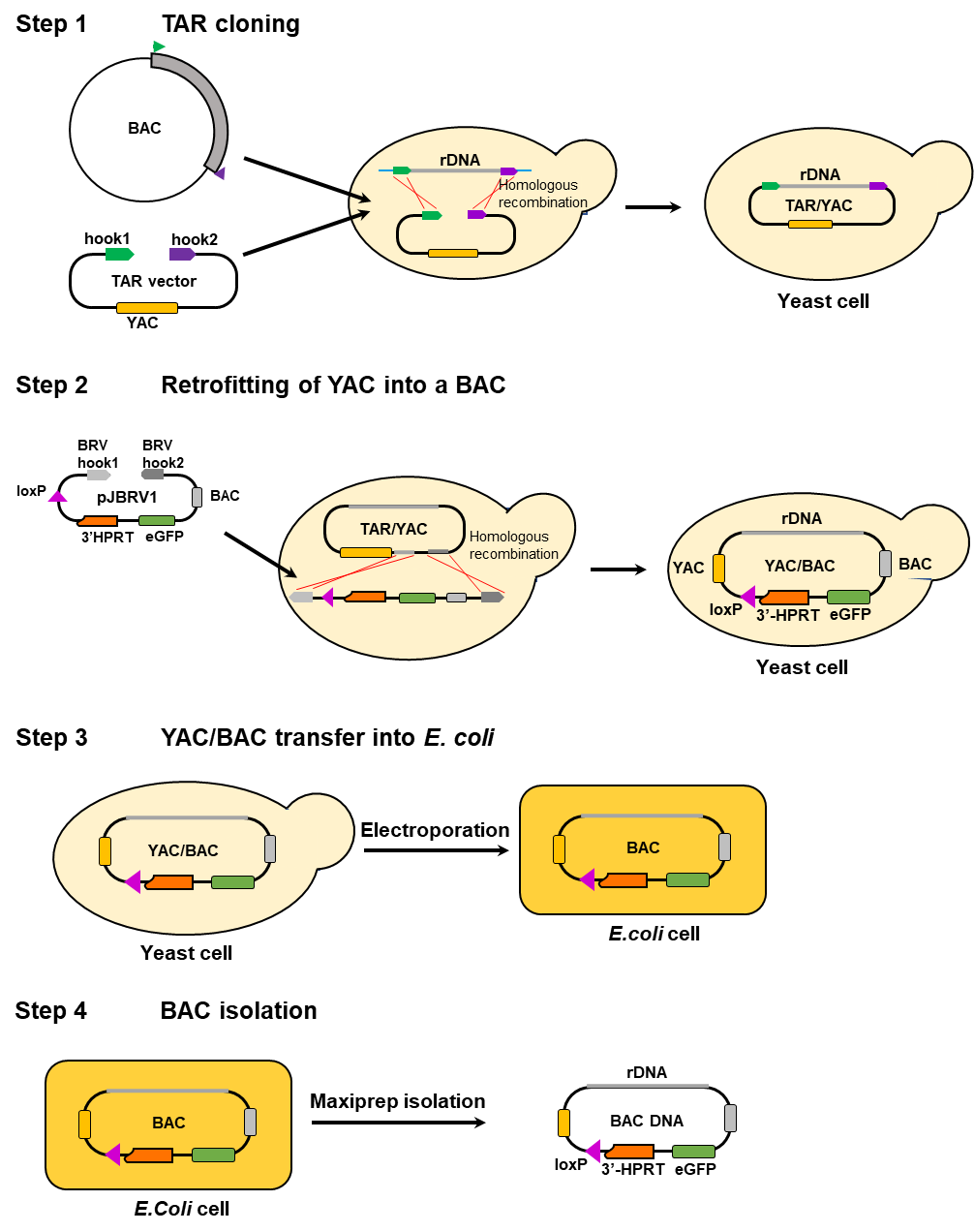


**Figure S1** Scheme of consecutive experimental steps from selective TAR isolation of the rDNA-containing regions of interest and distal and proximal junction (DJ and PJ) sequences in yeast *S. cerevisiae* in a YAC form to isolation of TAR clones in a BAC form from *E. coli* cells. **Step 1:** TAR isolation of either the complete rDNA unit, its transcribed part (45S), non-transcribed part (IGS) or the DJ sequence from RP11-337M7 BAC DNA (Ac. No. AL592188) or the PJ sequence from JH42 BAC DNA (Ac. No. MT497460) with the TAR vectors. Each TAR vector contains two targeting sequences (hook1 in green and hook2 in purple), a YAC cassette [a yeast centromeric locus (*CEN*) and a yeast selectable marker *HIS3*]. Before TAR cloning, TAR vectors are linearized between the hooks to make them highly recombinogenic. Homologous recombination between the targeting hooks in a TAR vector and the targeted sequences in a BAC DNA leads to the rescue of a region of interest as a circular TAR/YAC molecule. **Step 2:** Retrofitting of the circular TAR/YAC molecules into BAC molecules using the pJBRV1 vector containing a 3′ HPRT-loxP-eGFP cassette. BamHI digestion of pJBRV1 expose BRV-hook 1 and BRV-hook2 sequences that have homology to the YAC cassette in the TAR/YAC molecules. Recombination of the hook sequences with a TAR/YAC molecule in yeast leads to replacement of the ColE1 origin of replication by the F′ factor origin of replication (a BAC cassette), allowing subsequent YAC/BAC propagation in a BAC form. **Step 3:** Transfer of YAC/BAC molecules from yeast to *E. coli* cells by electroporation. **Step 4:** BAC DNA isolation.

**
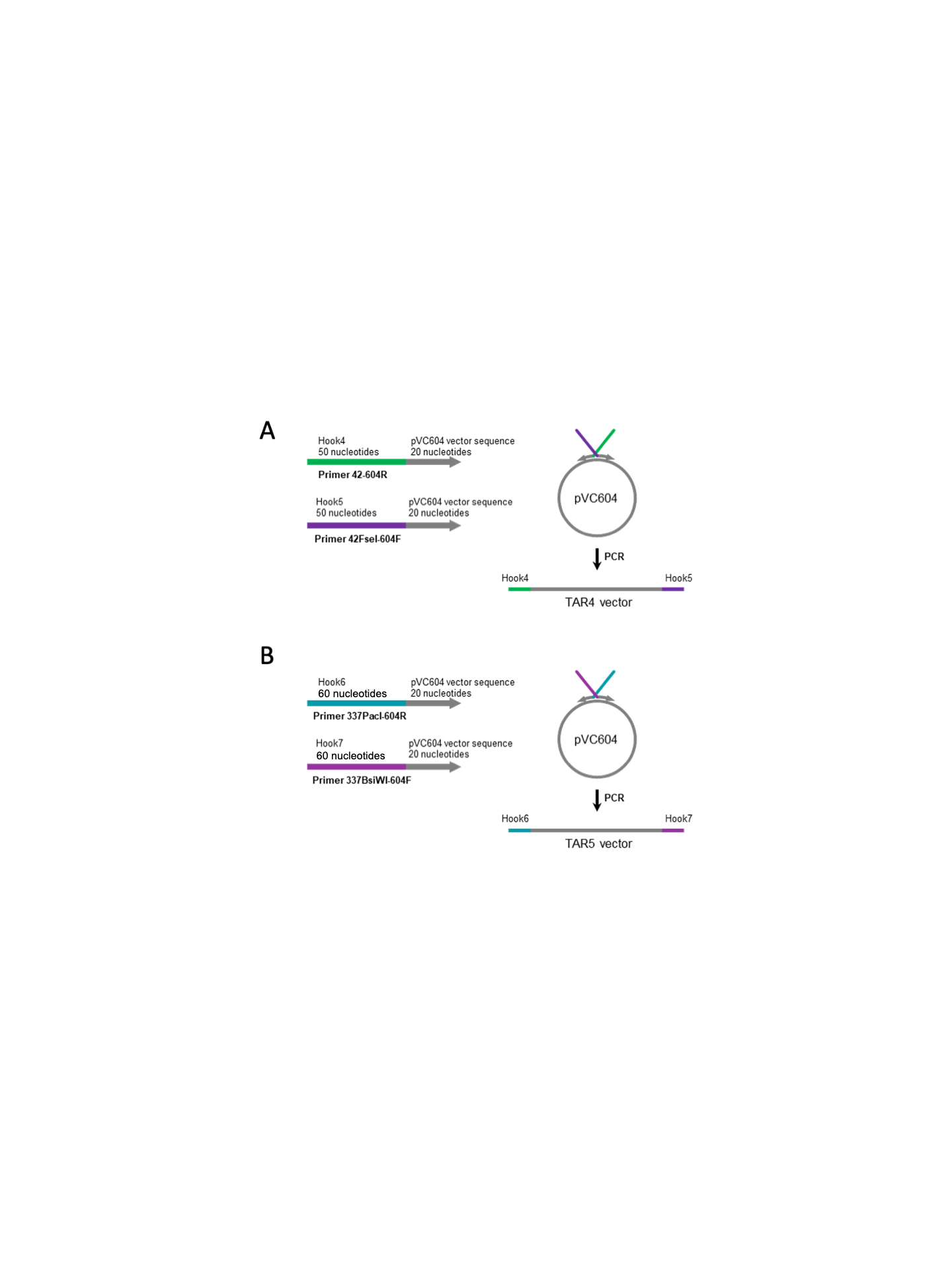
**

**Figure S2** Construction of TAR4 vector (**A**) used to clone the PJ sequence and TAR5 vector (**B**) used to isolate the DJ sequence. The vectors were prepared by PCR amplification reaction (Gibson et al 2008) using the corresponding primer pairs as illustrated (42FseI-604F/42-604R and 337PacI-604R/337BsiWI-604F) (Supplementary Table S1). 42FseI-604F/42-604R primers contain hook4 and hook5 (in green and in purple). 337PacI-604R/337BsiWI-604F primers contain hook6 and hook7 (in green). Each primer has a 20 bp sequence homologous to the pVC604 vector (in brown). After PCR reaction, linear TAR4 and TAR5 vectors with the exposed targeting sequences (hooks) are developed. The linear TAR vectors, after gel purification, were used in TAR cloning experiments.

**
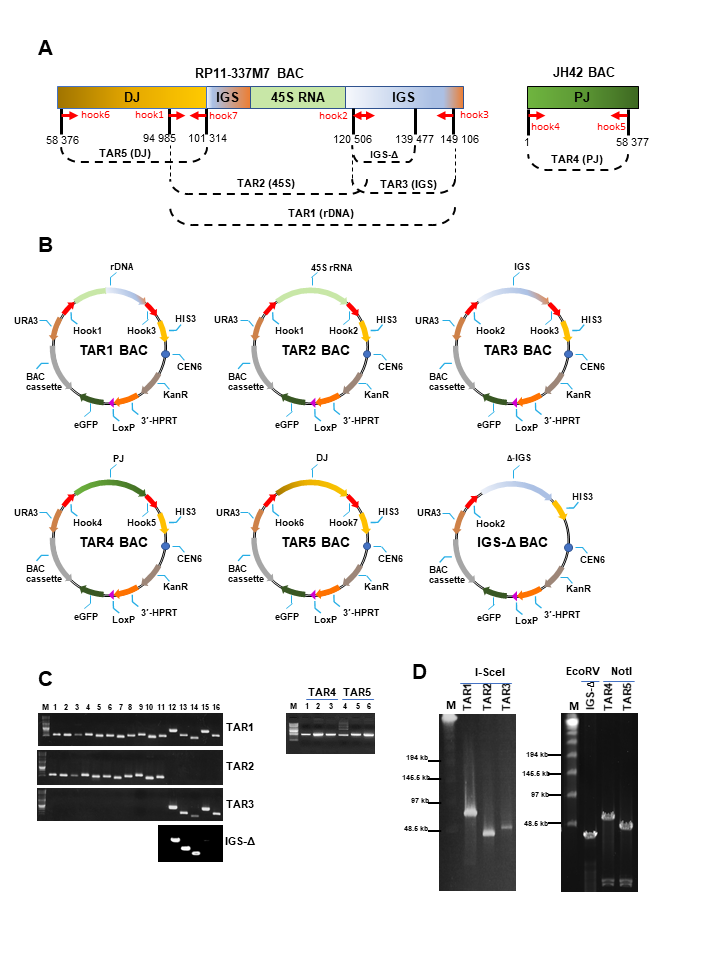
**

**Figure S3** TAR constructs and their physical characterization. (**A)** TAR1 (rDNA) construct was isolated by TAR1 vector containing hook1 and hook3. TAR2 (45S) construct was isolated by TAR2 vector containing hook1 and hook2. TAR3 (IGS) construct was isolated by TAR3 vector containing hook2 and hook3. TAR4 (PJ) construct was isolated by TAR4 vector containing hook4 and hook5. TAR5 (DJ) construct was isolated by TAR 5 vector containing hook6 and hook7. The IGS-D construct was obtained by deletion of the 3’ end of the IGS sequence (this part includes pre-promoter sequences involved in regulation of rRNA transcription). (**B)** Schemes of TAR/BAC constructs. TAR1 construct contains the entire rDNA unit (rDNA). TAR2 construct contains the transcribed part of the rDNA unit (45S). TAR3 construct contains the IGS sequence. TAR4 construct contains the PJ sequence. TAR5 construct contains the DJ sequence. The IGS-Δ construct contains the 3’-end of the IGS sequence. Each construct has YAC and BAC cassettes. A YAC cassette includes a yeast centromeric locus CEN6 and a yeast selectable marker HIS3. A BAC cassette includes the F′ factor origin of replication. Each construct has a 3′ HPRT-loxP-GFP sequence consisting of the mammalian color marker GFP, a loxP loading site and a 3’-part of the HPRT gene, the kanamycin resistant gene Kan^R^ encoding a phosphotransferase and a yeast selectable marker URA3. (**C)** PCR analysis of BAC DNA of different constructs. PCR analysis of the TAR1 construct with a set of diagnostic primer pairs for 18S, 5.8S, 28S and IGS (lanes 1-16) (Table S1). PCR analysis of the TAR2 construct with a set of diagnostic primer pairs for 18S, 5.8S and 28S (lanes 1-11) (Table S1). PCR analysis of the TAR3 construct with a set of diagnostic primer pairs for the IGS sequence (lanes 12-16) (Table S1). PCR analysis of the IGS-D construct with a set of diagnostic primers for the IGS sequence (lanes 12, 13, 14) (Table S1). PCR analysis of the TAR4 construct with a set of diagnostic primers for the left and right junctions and the internal part of the PJ sequence (lanes 1, 2, 3). PCR analysis of the TAR5 construct with a set of diagnostic primers for the left and right junctions and the internal part of the DJ sequence (lanes 4, 5, 6). M - Quick-Load 1 kb Plus DNA Ladder (New England BioLabs). (**D)** CHEF analysis of BAC DNA of the TAR1, TAR2, TAR3, TAR4, TAR5 and IGS-D constructs. BAC DNA of the TAR1, TAR2 and TAR3 constructs was linearized by I-SceI. BAC DNA of TAR4 and TAR5 constructs was digested by NotI. BAC DNA of the IGS-D construct was digested by EcoRV. M – a marker [CHEF DNA Size Lambda Ladder (BIO-RAD)].

**
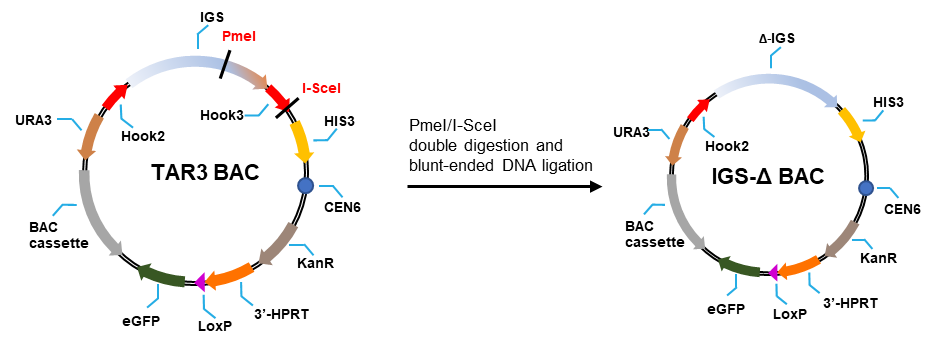
**

**Figure S4** The construct IGS-Δ was obtained by ligation of the PmeI/I-SceI digested TAR3 BAC construct (IGS). The IGS-Δ construct contains a 18,971 bp fragment corresponding to the IGS sequence deleted by 9,630 bp at the 3’ end of IGS. The basic part of the TAR3 BAC and IGS-Δ BAC constructs contain BAC (F’ origin of replication) and YAC [the HIS3 marker and a centromere from the yeast chromosome 6 (CEN6)] cassettes. The constructs also contain the mammalian color eGFP marker, a loxP loading site and the 3’-part of the HPRT gene.

**
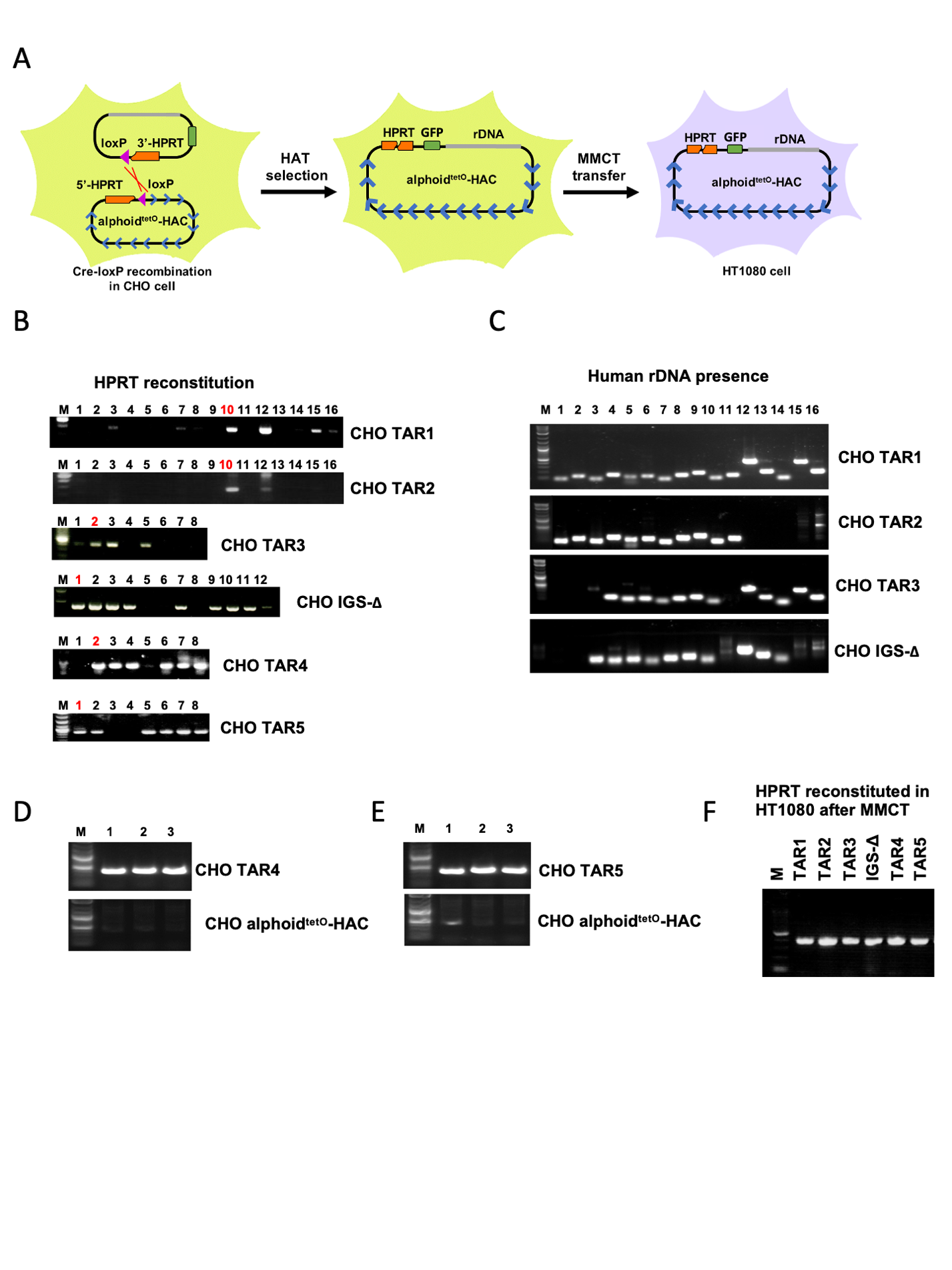
**

**Figure S5** Scheme of consecutive experimental steps from TAR constructs insertion into the alphoid^tetO^-HAC vector in hamster CHO cells to further HACs transfer to human HT1080 cells. (**A)** The BAC DNA constructs were loaded into a unique loxP site of the alphoid^tetO^-HAC by Cre-loxP recombination system in hamster CHO cells. The alphoid^tetO^-HACs carrying different constructs were MMCT transferred from CHO cells into human HT1080 cells. (**B**) Loading of the constructs into the loxP site of the HAC is accompanied by reconstruction of the HPRT gene allowing cell selection on the HAT medium. PCR analysis of the clones after insertion of TAR1 BAC (16 clones), TAR2 BAC (16 clones), TAR3 BAC, (8 clones), TAR4 BAC (8 clones), TAR5 BAC (8 clones) and IGS-BAC (12 clones) constructs into the loxP site. PCR products were sequenced to confirm reconstitution of the HPRT gene. Clones picked up for further analysis are marked in red. (**C-E**) PCR analysis of different constructs in hamster CHO cells. (**C**) PCR analysis of the TAR1 construct (clone 10) with a set of diagnostic primer pairs for 18S, 5.8S, 28S and IGS (lanes 1-16) (Table S1). PCR analysis of the TAR2 construct (clone 10) with a set of diagnostic primer pairs for 18S, 5.8S and 28S (lanes 1-11) (Table S1). PCR analysis of the TAR3 construct (clone 2) with a set of diagnostic primer pairs for the IGS sequence (lanes 12-16) (Table S1). PCR analysis of the IGS-Δ construct (clone 1) with a set of diagnostic primers for the IGS sequence (lanes 12, 13, 14) (Table S1). (**D**) PCR analysis of the TAR4 construct (clone 2) with a set of diagnostic primers for the left and right junctions and the internal part of the PJ sequence (lanes 1, 2, 3) (Table S1). (**E**) PCR analysis of the TAR5 construct (clone 1) with a set of diagnostic primers for the left and right junctions and the internal part of the DJ sequence (lanes 1, 2, 3) (Table S1). (**F)** PCR analysis of the HACs carrying different constructs with HPRT diagnostic primers (Table S1) after MMCT transfer of HACs from hamster CHO cells to human HT1080 cells. M - Quick-Load 1 kb Plus DNA Ladder (New England BioLabs).

**
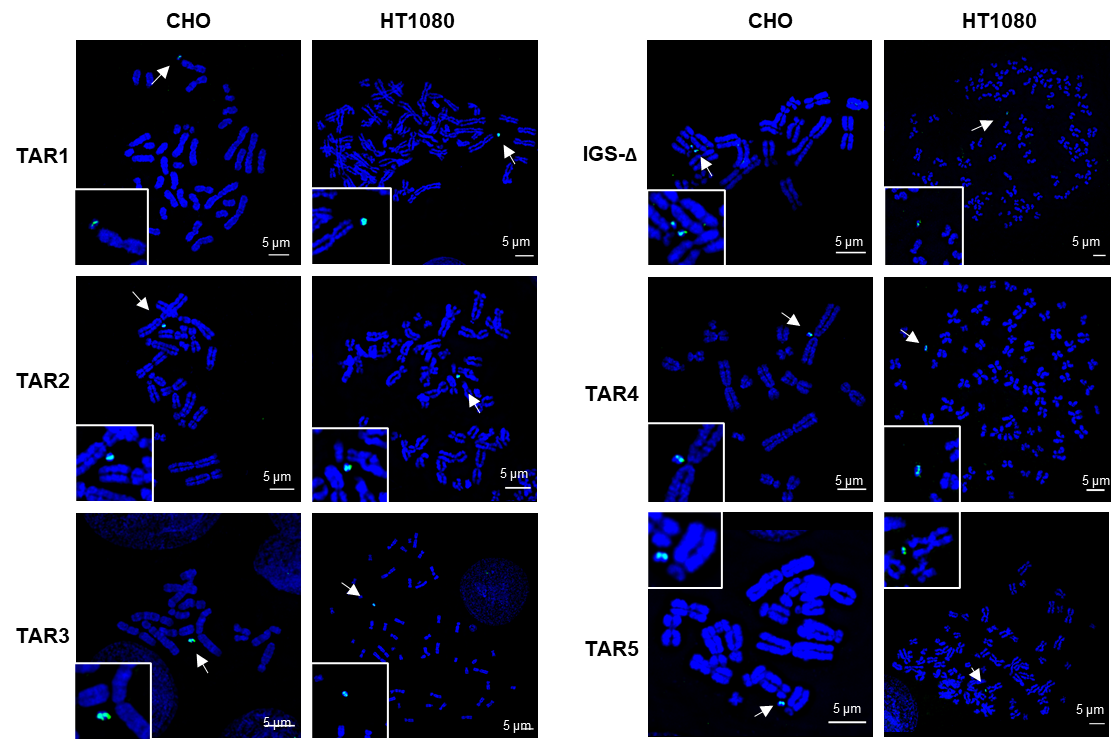
**

**Figure S6** FISH analysis of the alphoid^tetO^-HAC carrying different constructs in hamster CHO and human HT1080 cells using a specific probe for the HAC vector (in green, indicated with a white arrow) (see Materials and methods). TAR1 construct – the entire rDNA unit (rDNA). TAR2 construct – the transcribed part of the rDNA unit (45S). TAR3 construct – the IGS sequence. IGS-Δ construct – the 3’-end of the IGS sequence. TAR4 construct – the PJ sequence. TAR5 construct – the DJ sequence.

.

**Figure S7** rDNA transcription in human/mouse hybrid cell lines A9(#13) 89-2 (chr13), A9 hygro14 10F (chr14), A9(Neo15)-3 (chr15), A9 #21-16 (chr21), A9#22(γ2) (chr22γ2) and A9HyTK-22 (chr22TK) containing human chromosomes 13, 14, 15, 21 and 22. Human ribosomal genes are transcriptionally silent in hybrid cell lines. Total RNA samples from human/mouse monochromosomal hybrid cells and human RPE cells were used in RP-qPCR with human-specific primers for 45S rRNA and GAPDH primers specific for both mouse and human. rRNA relative expression in A9(#13) 89-2 (chr13) cell line is equal to one.

**
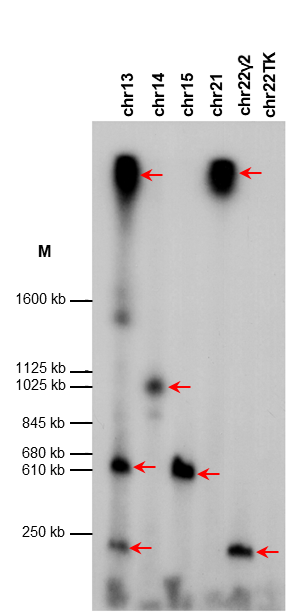
**

**Figure S****8** Southern blot analysis of six human/mouse hybrid cell lines A9(#13) 89-2 (chr13), A9 hygro14 10F (chr14), A9(Neo15)-3 (chr15), A9 #21-16 (chr21), A9#22(γ2) (chr22γ2) and A9HyTK-22 (chr22TK) containing human chromosomes 13, 14, 15, 21, and 22. Genomic DNA possessing one of the acrocentric chromosomes was isolated from each hybrid cell line, digested by EcoRV and separated by CHEF gel electrophoresis with range 250 kb-2.5 Mb. The rDNA repeats were detected with a radioactively labeled probe specific to the rDNA intergenic spacer (IGS) (see Materials and Methods). M - CHEF DNA Size Marker, 0.2–2.2 Mb, *S. cerevisiae* Ladder (Bio-Rad, cat. no. 1703605).


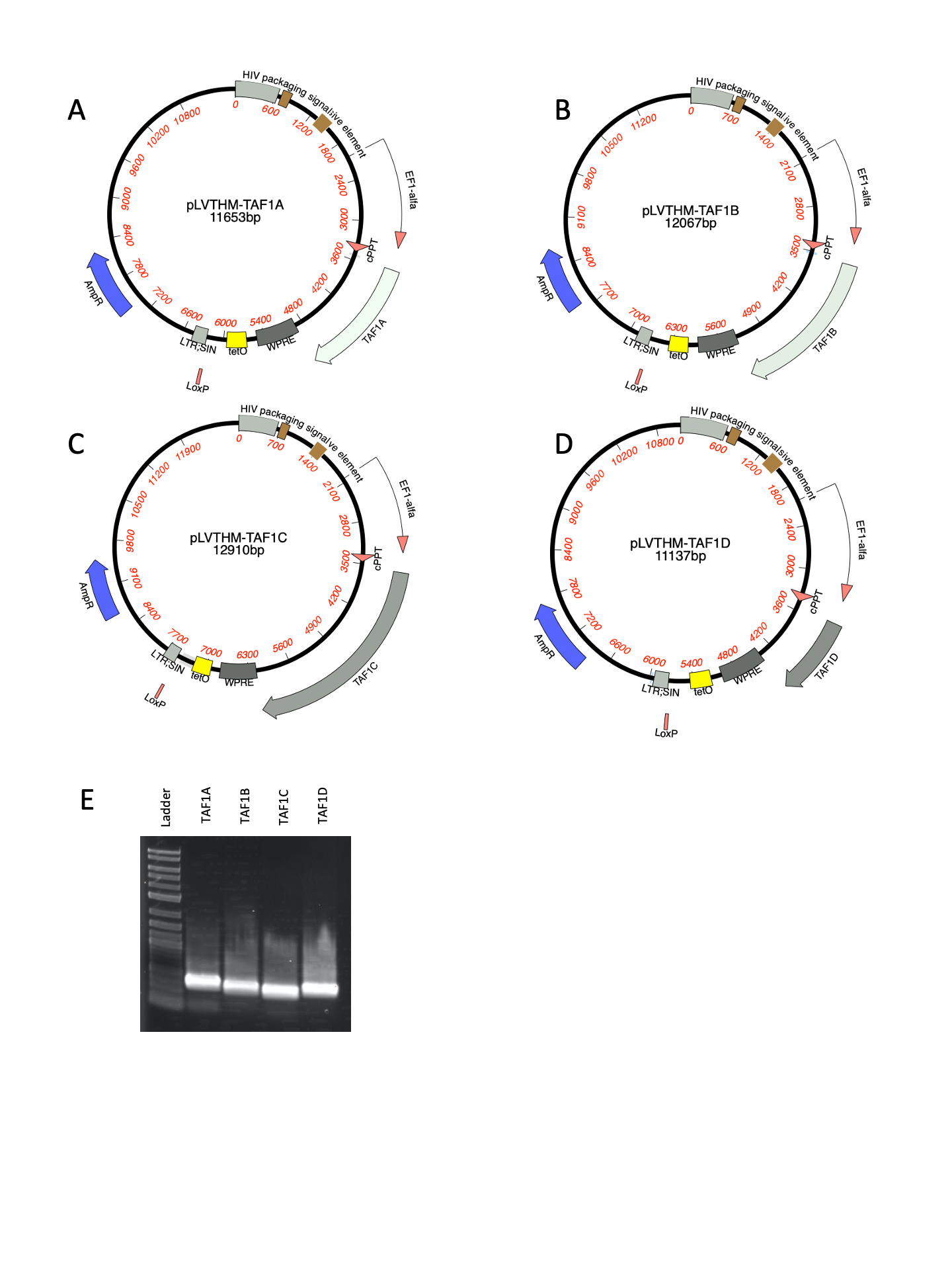


**Figure S9** Expression of lentiviral vectors encoding TAF1A, TAF1B, TAF1C, and TAF1D. (**A-D**) Schemes of four pLVTHM vectors carrying TAF1A, TAF1B, TAF1C, and TAF1D transcription factors. (**E**) RT-PCR confirming expression of TAF1A, TAF1B, TAF1C, and TAF1D after infection of A9#13 89-2 cells by the lentiviral vectors.

**
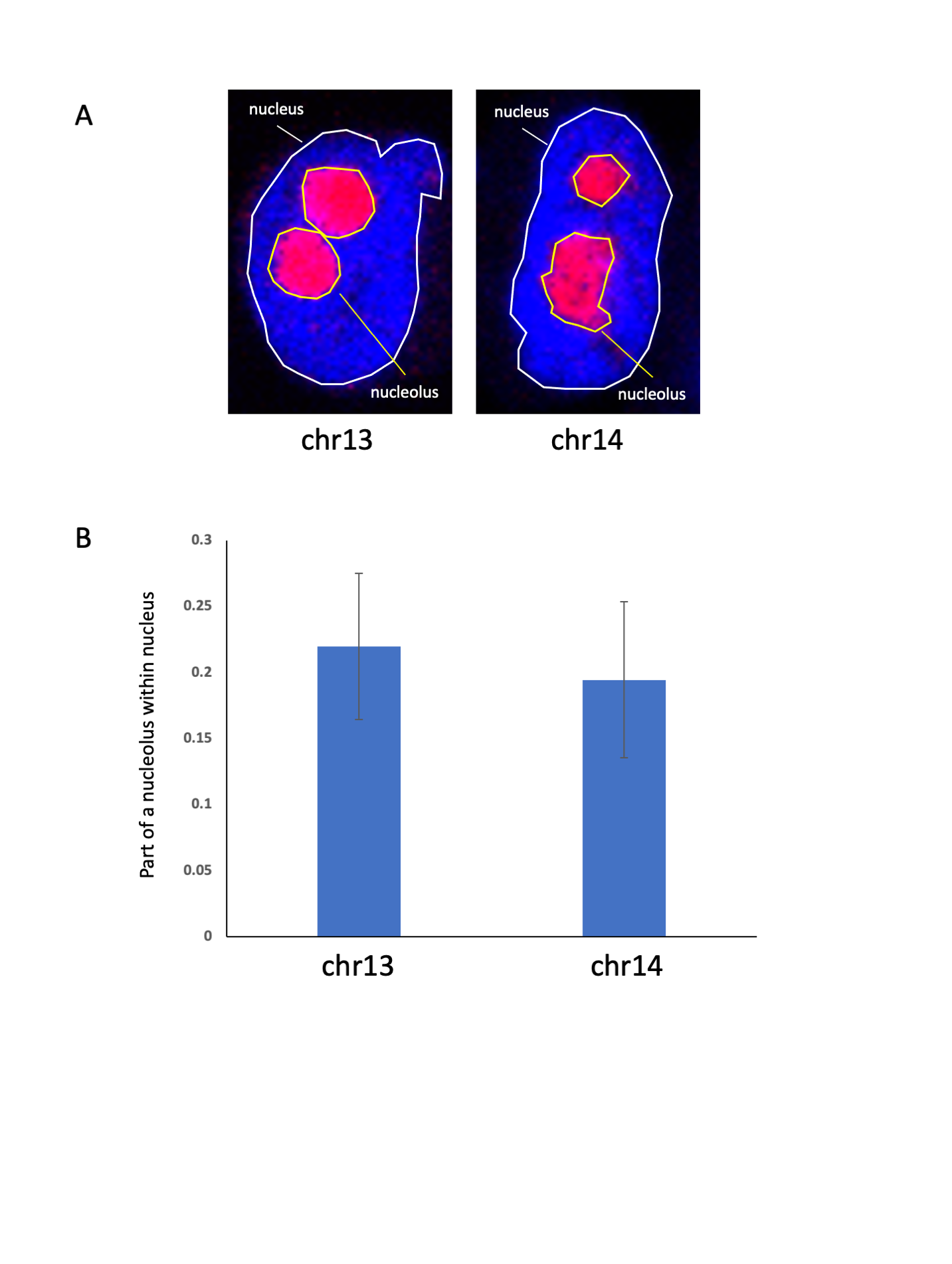
**

**Figure S10**Measurement of the nucleolus area within the nucleus. (**A**) An example of the analyzed images. Blue -DAPI staining. Red- RRP1antibody staining. White line – a nucleus border. Yellow line- a nucleolus border. (**B)** Comparison of a nucleolus area within a nucleus between human/mouse monochromosomal hybrid cell lines A9(#13) 89-2 containing chromosome 13 and A9 hygro14 10F containing chromosome 14. No statistically significant differences were observed by using two-tailed nonparametric Mann-Whitney u test.

**
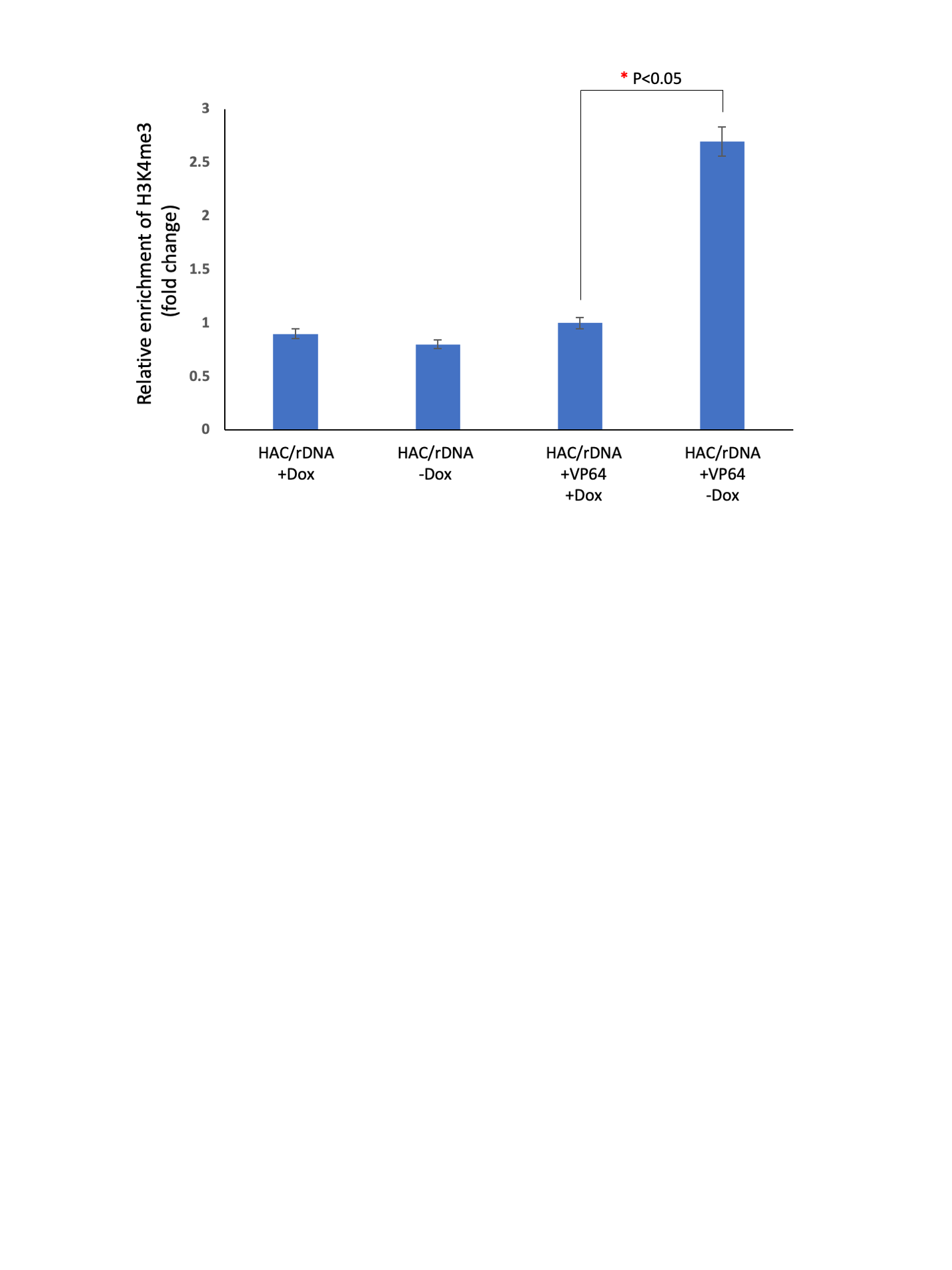
**

**Figure S11** Chromatin immunoprecipitation (ChIP) of non-coding tetO-alpha-satellite DNA repeats in the alphoid^tetO^-HAC carrying whole rDNA unit using antibodies against H3K4me3 in the cells growing in doxycycline-containing medium compared to that of the cells that have been grown in the medium lacking doxycycline after 7 days of culture after transfection by the tetR-tTA^VP64^ fusion protein genes. Data were normalized to the internal 5S rDNA controls. Error bars indicate SD. Significant differences were calculated using two-tailed nonparametric Mann-Whitney u test.

**
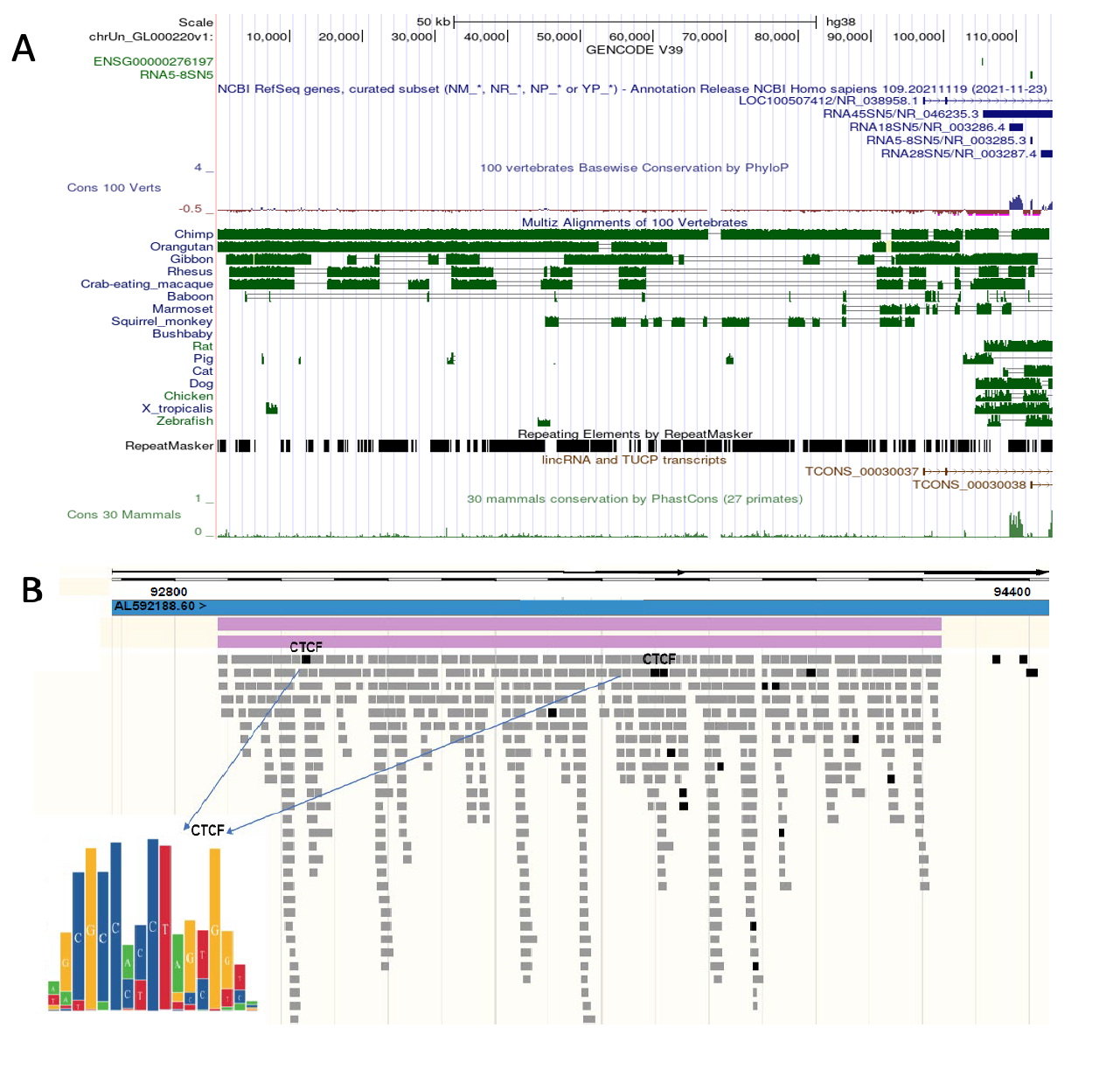
**

**Figure S12** The structure of the proximal 100 kb sequence of the human DJ region. (**A**) Schematic representation of the proximal end of the human DJ region (IDs: GL000220 and AL592188.60) and its comparative analysis, based on UCSC genomic data and multiple alignment of 100 mammalian genomes (shown in green; track “Multiz Alignments of 100 vertebrates” on https://genome.ucsc.edu/). Shown are 9 primate and 7 vertebrate genomes with the best unambiguous levels of similarity. The PhyloP conservation profile generated for 100 vertebrates (blue and red; track “PhyloP” in UCSC) and the PhastCons conservation profile for 30 mammals (green; track “PhastCons” in UCSC) are shown separately. Repeats elements predicted by Repeat Masker are shown in black. (**B**) Overview of predicted TF sites with detailed images for the 92.5-94.5 kb region of AL592188.60, which is located in close proximity to the rDNA array. Regions with predicted (gray boxes) and experimentally confirmed (black boxes) TF binding sites (shown in pink) are mapped to the 92.5-94.5 kb region upstream of LncRNA NR_038958. Two CTCF sites are marked, and the CTCF consensus is shown in the inset.

**
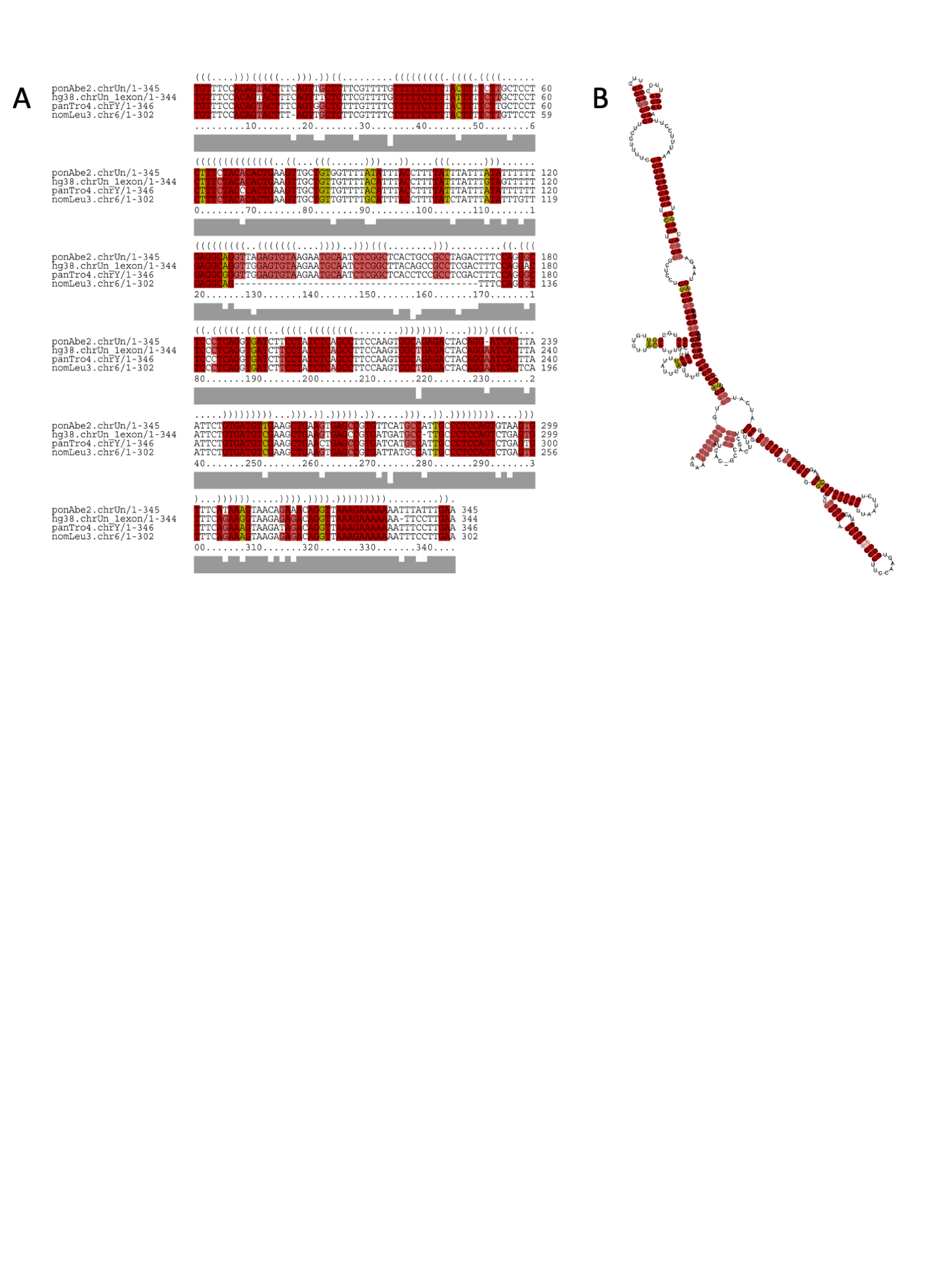
**

**Figure S13** Consensus folding for multiple alignment of the 5’end of predicted LncRNA NR_038958 in four primate species.

A


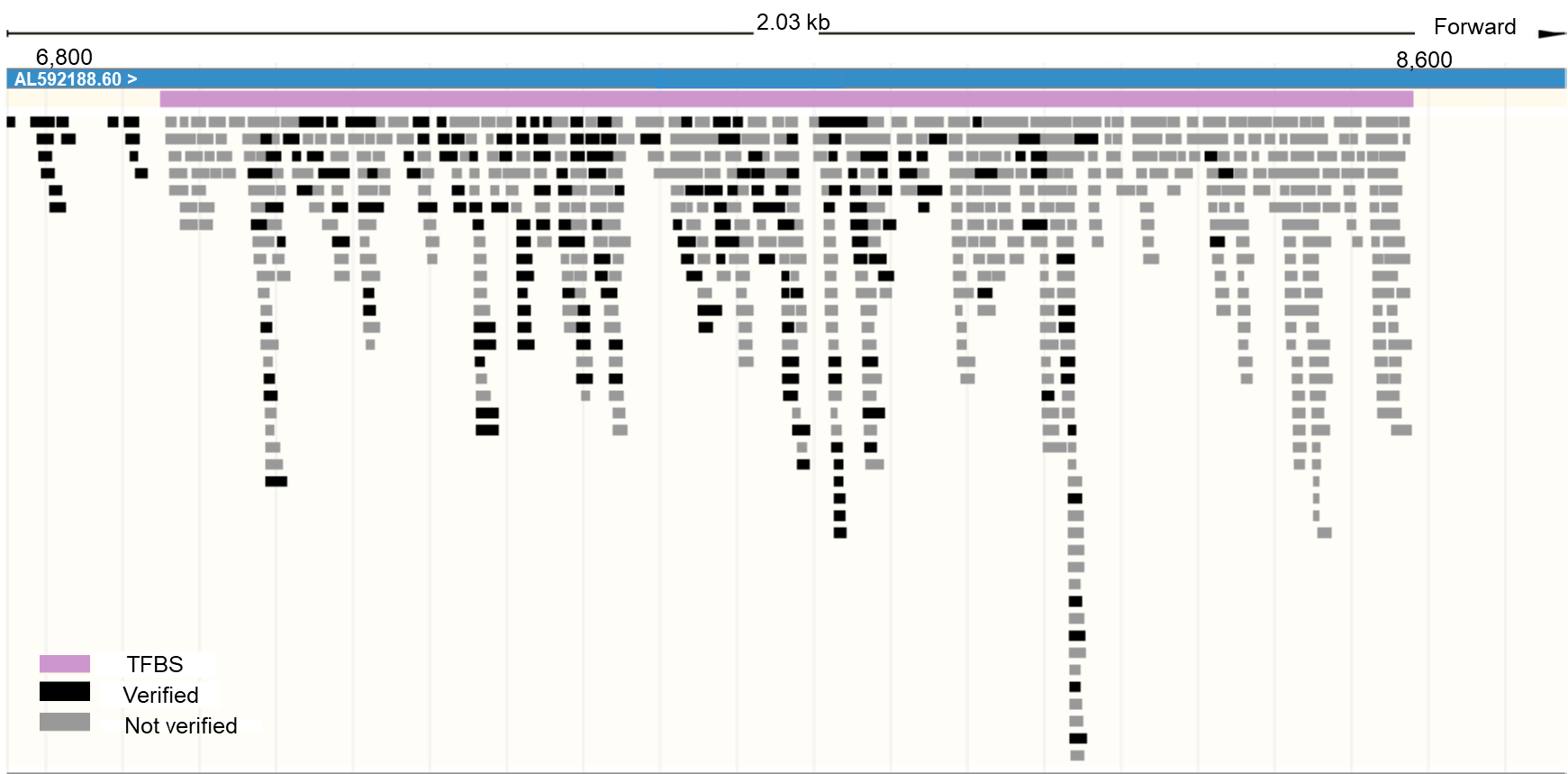


B

**
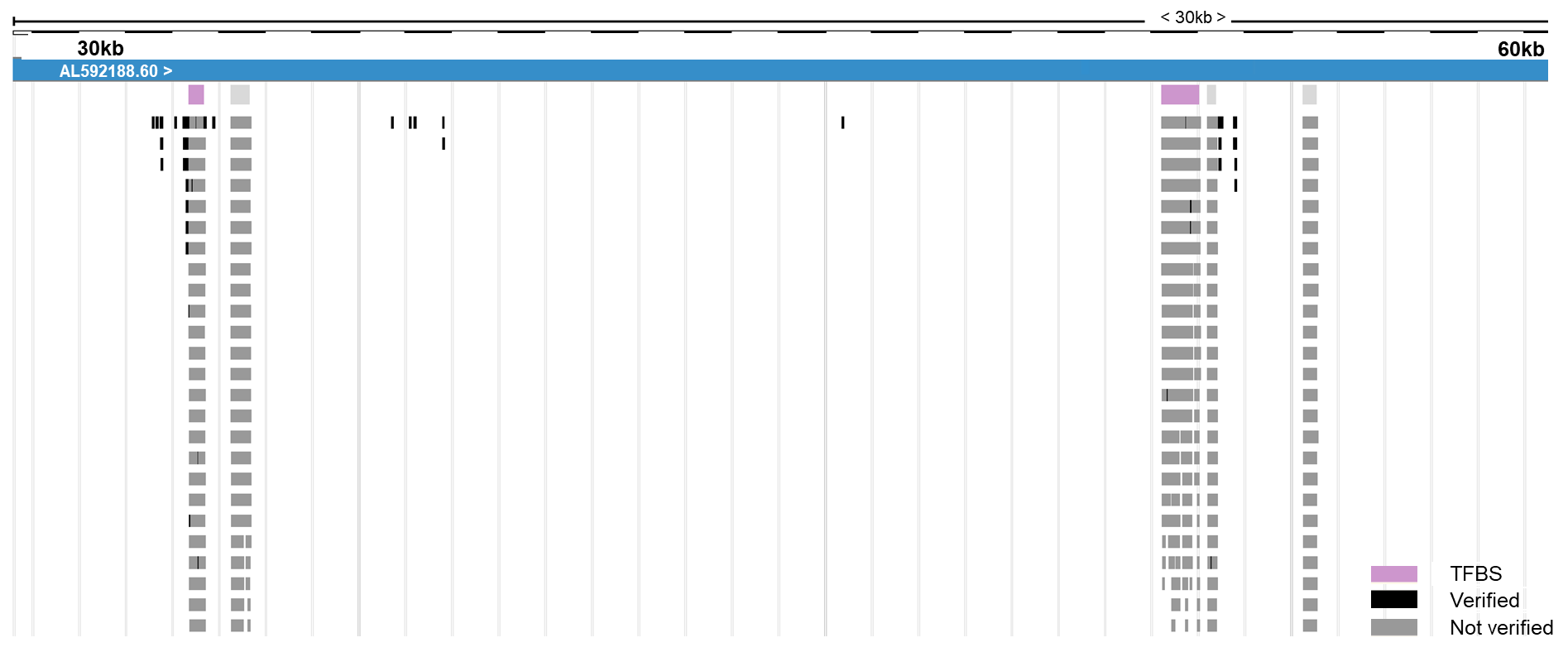
**

**Figure S14** Three conserved sequences enriched with potential TF sites for protein binding are located near the 6-8 kb (**A)**, and between the 32- 50 kb regions (**B**).


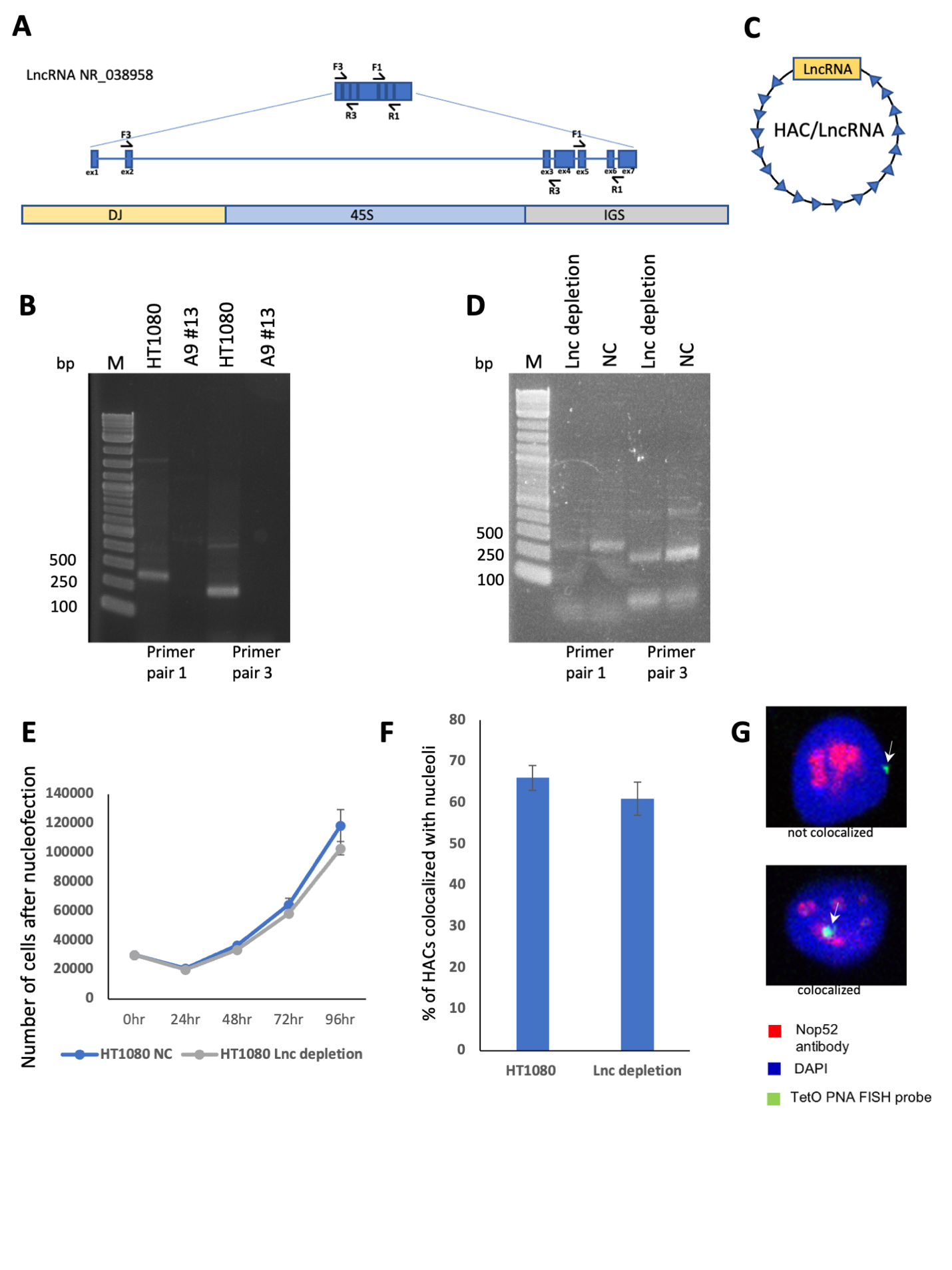


**Figure S15** Expression and depletion of LncRNA NR_038958. (**A**) Schematic representation of LncRNA NR_038958 with 7 annotated exons (blue boxes). (**B**) RT-PCR confirming the existence of the spliced LncRNA transcript in human HT1080 cells and absence of expression in the human/mouse hybrid A9#13 89-2. Primer pair 1 - F1/R1. Primer pair 3 - F3/R3. (**C**) Scheme of the HAC vector carrying the LncRNA NR_038958 insert (HAC/LncRNA). (**D**) RT-PCR confirming a partial depletion of LncRNA transcript in HT1080 cells. NC - a negative control (untreated cells). (**E**) Growth curves of HT1080 cells after LncRNA depletion (a gray curve) vs. untreated cells (a blue curve). NC - a negative control (untreated cells). HT1080 cells were electroporated with antisense oligos (see Materials and method). Three independent experiments were performed. (**F**) Quantification of 3D immune-FISH analysis. The percentage of cells carrying the HAC/LncRNA associated with nucleoli before and after LncRNA depletion. (**G**) Representative images of 3D immuno-FISH. Anti-RRP1 antibodies (Nop52) (red) used to visualize nucleoli were combined with a BAC FISH probe containing the tetO sequence of the HAC (green). Nuclei were stained with DAPI (blue). Nucleolar- and non-nucleolar-associated HACs are indicated by white arrowheads.

**Table S1 List of primers used in this work**

**______________________________________________________________________________**

**Primers for hook amplification**

**______________________________________________________________________________**

TAR vector 1 (rDNA unit plus 7.5 kb DJ)

______________________________________________________________________________

Hook 1

3 5' hook rDNA-BamHI-F1 5’-ATGCAGGATCCAAAATCATGAACCGCTCACC-3’

38 5' hook rDNA-SalI-R1 5’-TCGTAGTCGACCTGGAGGCACAATAGCTCAC-3’

Hook 3

39 3' hook rDNA-SalI-F1 5’-ATGCAGTCGACGGTCCCTCTGCCGCGATCCTT-3’

117 3' hook rDNA-I-SceI-XhoI-R1 5’-TCGTACTCGAGATTACCCTGTTATCCCTACCCCTCCTACGACCGGGACAC-3’

______________________________________________________________________________

TAR vector 2 (external transcribed spacer ETS plus 7.5 kb DJ)

______________________________________________________________________________

Hook 1

37 5' hook rDNA-BamHI-F1 5’-ATGCAGGATCCAAAATCATGAACCGCTCACC-3’

38 5' hook rDNA-SalI-R1 5’-TCGTAGTCGACCTGGAGGCACAATAGCTCAC-3’

Hook 2

41 3' hook rDNA TU-SalI-F1 5’-ATGCAGTCGACAAAACTATGTAAATGATATTTCC-3’

118 3' hook rDNA TU-I-SceI-XhoI-R1 5’-TCGTACTCGAGATTACCCTGTTATCCCTACACGTGTATGTAAATGAAATGGG-3’

______________________________________________________________________________

TAR vector 3 (intergenic spacer IGS)

______________________________________________________________________________

Hook 2

43 5' hook rDNA SP-BamHI-F1 5’-ATGCAGGATCCAAAACTATGTAAATGATATTTCC-3’

44 5' hook rDNA SP-SalI-R1 5’-TCGTAGTCGACCACGTGTATGTAAATGAAATGGG-3’

Hook 3

39 3' hook rDNA-SalI-F1 5’-ATGCAGTCGACGGTCCCTCTGCCGCGATCCTT-3’

117 3' hook rDNA-I-SceI-XhoI-R1 5’-TCGTACTCGAGATTACCCTGTTATCCCTACCCCTCCTACGACCGGGACAC-3’

______________________________________________________________________________

**Detection primers**

**______________________________________________________________________________**

TAR vector 1 (rDNA unit plus 7.5 kb DJ)

______________________________________________________________________________

*5’ junction of vector with insert*

M13-F 5’-TGTAAAACGACGGCCAGT-3’

48 5' hook rDNA seq-R1 5’-GCCTCTAAGCGAGACTCCA-3’

*3’ junction of vector with insert*

92 3' hook rDNA seq-F2 5’-CGATCCTTTCTGGCGAGTCC-3’

M13-R 5’-CAGGAAACAGCTATGACC-3’

TAR vector 2 (external transcribed spacer ETS plus 7.5 kb DJ)

______________________________________________________________________________

*5’ junction of vector with insert*

M13-F 5’-TGTAAAACGACGGCCAGT-3’

48 5' hook rDNA seq-R1 5’-GCCTCTAAGCGAGACTCCA-3’

*3’junction of vector with insert*

52 3' hook rDNA TU seq-F1 5’-ATCTTATTTTCTGAACGCTGCC-3’

M13-R 5’-CAGGAAACAGCTATGACC-3’

______________________________________________________________________________

TAR vector 3 (intergenic spacer IGS)

______________________________________________________________________________

*5’junction of vector with insert*

M13-F 5’-TGTAAAACGACGGCCAGT-3’

55 5' hook

*3’ junction vector with insert*

92 3' hook rDNA seq-F2 5’-CGATCCTTTCTGGCGAGTCC-3’

M13-R 5’-CAGGAAACAGCTATGACC-3’

______________________________________________________________________________

**Primers for rDNA unit**

______________________________________________________________________________

**18S**

rDNA 105734-1F 5’-TGTCAGGCGTTCTCGTCTC-3’

rDNA 105734-1R 5’-GACGTCACCACATCGATCAC-3’

rDNA 106285-2F 5’-GAACGGTGGTGTGTCGTTC-3’

rDNA 106285-2R 5’-GCGTCTCGTCTCGTCTCAC-3’

rDNA 109524-4F 5’-AAACGGCTACCACATCCAAG-3’

rDNA 109524-4R 5’-CCTCCAATGGATCCTCGTTA-3’

**______________________________________________________________________________**

**5.8S**

rDNA 112056-5F 5’-GTCGATGAAGAACGCAGCTA-3’

rDNA 112056-5R 5’-GACGCTCAGACAGGCGTAG-3’

______________________________________________________________________________

**28S**

rDNA 113431-6F 5’-AGTAACGGCGAGTGAACAGG-3’

rDNA 113431-6R 5’-GCCTCGATCAGAAGGACTTG-3’

rDNA 113678-7F 5’-GCTAAATACCGGCACGAGAC-3’

rDNA 113678 -7R 5’-TTCACGCCCTCTTGAACTCT-3’

rDNA 115727-8F 5’-GAACTTTGAAGGCCGAAGTG-3’

rDNA 115727-8R 5’-ATCTGAACCCGACTCCCTTT-3’

rDNA 117701-9F 5’-TGGGTTTTAAGCAGGAGGTG-3’

rDNA 117701-9R 5’-GACGGTCTAAACCCAGCTCA-3’

rDNA 118316-10F 5’-CCATTCGTAGACGACCTGCT-3’

rDNA 118316-10R 5’-CTTGTGTCGAGGGCTGACTT-3’

_____________________________________________________________________________

**IGS (spacer)**

rDNA 119957-11F 5’-CGGAACTCCCTCTCCTACATT-3’

rDNA 119957-11R 5’-AAGAAAACGCAAGGCAAAA-3’

rDNA 126263-11F 5’-GTGTTCCCGTGAGGAGTGAT-3’

rDNA 126263-11R 5’-ACAGAGAGAAGGCCCTAGCC-3’

rDNA 126263-12F 5’-GTGTTCCCGTGAGGAGTGAT-3’

rDNA 126263-12R 5’-ACAGAGAGAAGGCCCTAGCC-3’

rDNA 134304 -13F 5’-CACCTAGCGGCCACTGTTAT-3’

rDNA 134304-13R 5’-AGTAAAGCGCCGATCAAAGA-3’

rDNA 138700-14F 5’-GAATGCGACTCCTGCAAAAT-3’

rDNA 138700-14R 5’-TTCCCAAGTCTGGTTGATCC-3’

rDNA 142197-15F 5’-GGGAGTCCGAGACAGAATGA-3’

rDNA 142197-15R 5’-AGCTCCTGTGGTTTCAGGTG-3’

rDNA 144016-15F 5’-AGGAGTCCCCTGGTCTGTCT-3’

rDNA 144016-15R 5’-GTCAAGGTCCAAACCGAAAA-3’ ______________________________________________________________________________

**Primers for construction of TAR4 and TAR5 vectors by PCR**

**______________________________________________________________________________**

TAR vector 4 (PJ) (low case letters-Hook4 and Hook5; capital case letters – vector part)

______________________________________________________________________________

42FseI-604F 5’-gttggcgcagagcggcgcggcggagacagcggcgacgggaatccggccggGCAAGGCGATTAAGTTGGG-3’

42-604R 5’-ctcaccttcccctcatggaccttctgactttctgcacctgctccggcgctACATCCCCCCTTCGCCAGC-3’

______________________________________________________________________________

TAR vector 5 (DJ) (low case letters-Hook6 and Hook7 capital case letters – vector part)

______________________________________________________________________________

337PacI-604R 5’-atttggctcttgtaatttgcagtggaaagcttatatgagtatctaactctaaaccaattaACATCCCCCCTTCGCCAGC-3’

337BsiWI-604F 5’-actcagggtgtttggggtcttctggggaaatgttctgaaacaatggagtattttggtctgGCAAGGCGATTAAGTTGGG-3’

______________________________________________________________________________

**Detection primers**

**______________________________________________________________________________**

TAR vector 4 (PJ)

*5’ junction of vector with insert*

RCO-504F 5’-GATTTAGAGCTTGACGGGGA-3’

42-detR 5’-TCACCTCTCCTCCCACAAACT-3’

*3’ junction of vector with insert*

RCO-502R 5’-TGGAGCTCCAATTCGCCCT-3’

42-detF 5’-CCACCTAACTGGCTCCCAAAC-3’

*Internal*

42-det IN F 5’-TCCTGAGGAAGCCTCTTTATTG-3’

42-det IN R 5’-CCAACCTCACATGGTCTGAA-3’

______________________________________________________________________________

TAR vector 5 (DJ)

______________________________________________________________________________

*5’ junction of vector with insert*

337 det-PacI-R 5’-GCACCACAAGCAGCAGTCATA-3’

RCO-504F 5’-GATTTAGAGCTTGACGGGGA-3’

*3’ junction of vector with insert*

337-det-BsiWI-F 5’-CTTTCATCACCACCACCGAAGA-3’

RCO-502R 5’-TGGAGCTCCAATTCGCCCT-3’

*Internal*

337-det IN F 5’-CCAGTGCAGAACCCATCTATT-3’

337-det IN R 5’-CCTGAGAAGTCTGTGTGTGTATG-3’

______________________________________________________________________________

**Primers for human rDNA copy numbers**

**______________________________________________________________________________**

*Human IGS*

rDNA 134304 -13F 5’-CACCTAGCGGCCACTGTTAT-3’

rDNA 134304-13R 5’-AGTAAAGCGCCGATCAAAGA-3’

rDNA 142197-15F 5’-GGGAGTCCGAGACAGAATGA-3’

rDNA 142197-15R 5’-AGCTCCTGTGGTTTCAGGTG-3’

*Mouse single copy*

Mouse Chr5 F 5’-GATCTGGCTTTCCGGCTATT-3’

Mouse Chr5 R 5’-CCCTATTTGTGTGGCCTCTT-3’

**Primers for human rRNA transcription**

**______________________________________________________________________________**

*Human 45S rRNA*

h45S-F 5’-CCGCGCTCTACCTTACCTACCT-3’

h45S-R 5’-GCATGGCTTAATCTTTGAGACAAG -3’

*Human/Mouse GAPDH*

GAPDH-F 5’-CCATCTTCCAGGAGGGAGA-3’

GAPDH-R 5’-GGCAGTGATGGCATGGACTGT -3’

______________________________________________________________________________

**Primers for Southern analysis**

**______________________________________________________________________________**

FOR-F 5’-GGGAGTCCGAGACAGAATGA-3’

REV-R 5’-AGCTCCTGTGGTTTCAGGTG-3’

______________________________________________________________________________

**Primers for HPRT gene reconstruction**

_____________________________________________________________________________

Lox137-R 5’-AGCCTTCTGTACACATTTCTTCTC-3’

Rev #6 5’-GCTCTACTAAGCAGATGGCCACAGAACTAG-3’

SV40 PA term rev 5’-AATGGTTACAAATAAAGCAATAGCATCAC-3’

______________________________________________________________________________

**Primers for lentiviral vectors construction**

______________________________________________________________________________

AscI_TAF1A 5’-TATGCAGGCGCGCCAATGAGTGATTTCAGTGAAGAATTAAAAGGGC-3’

SpeI_TAF1A 5’-TATGCAGGACTAGTTCAGAGTCTTGGATTTACAATACTGTATTTT-3’

AscI_TAF1B 5’-TATGCAGGCGCGCCAATGGACCTCGAGGAGGCGGAA-3’

SpeI_TAF1B 5’-TATGCAGGACTAGTTCAATGTCGTCTCACTTTCTTGGATCTTG-3’

AscI_TAF1C 5’-TATGCAGGCGCGCCAATGGACTTCCCCAGCTCCCTCCG-3’

SpeI_TAF1C 5’-TATGCAGGACTAGTTCAGAAGCCCATTCGAGGCTTCTTCC-3’

AscI_TAF1D 5’-TATGCAGGCGCGCCAATGGATAAATCAGGAATAGATTCTCTTGACC-3’

SpeI_TAF1D 5’-TATGCAGGACTAGTTCACATTTTCAGGCCTCTCTGTCCAGTA-3’

______________________________________________________________________________

**Primers for RT-PCR**

______________________________________________________________________________

rt-WRRE-R1 5’-GCCATACGGGAAGCAATAGCA-3’

TAF1A-F1 5’-AAACTGGTGGCCAGGCTTTC-3’

TAF1B-F1 5’-ACACATGTGACAACCTATGAAGAA-3’

TAF1C-F1 5’-TGCTCCGTGACTACATGGC-3’

TAF1D-F1 5’-GCAGAGGATGAGGATGCAACA-3’

NR_038958_F1 5’-TTGGGGCACACAGATGAGAC-3’

NR_038958_R1 5’-AATGGAGAGAAGCCCAAGGC-3’

NR_038958_F3 ﻿ 5’-ATAGAGACAAGGTAGCTCCA-3’

NR_038958_R3 ﻿ 5’-TCTGGAGCCTAAAAGTTCGA-3’

**Primers for ChIP**

______________________________________________________________________________

5S ribosome F1 5’-ACGCTGGGTTCCCTGCCGTT-3’

5S ribosome R1 5’-TGGCTGGCGTCTGTGGCACCCGCT-3’

tet-1 5’-CCACTCCCTATCAGTGATAGAGAA-3’

tet-3 5’-TCGACTTCTGTTTAGTTCTGTGCG-3’

______________________________________________________________________________

Modified antisense oligos for nucleofection.

______________________________________________________________________________

400 5’-mG*mA*mA*mA*mC*T*C*C*G*T*C*G*T*G*C*mC*mC*mA*mC*mG-3’

**Table S2 Number of human rDNA copies in human/mouse monochromosomal hybrid cells determined by qPCR**

| Cell line | rDNA copy number | 95% CI |
| --- | --- | --- |
| A9(#13) 89-2 (chr13) | 78 | 48-108 |
| A9hygro14 (chr14) | 39 | 26-53 |
| A9(Neo15)-3 (chr15) | 17 | 14-19 |
| A9 #21-16 (chr21) | 43 | 29-57 |
| A9#22 (γ2) (chr22γ2) | 9 | 7-11 |
| A9HyTK-22 (chr22TK) | 2 | 2 |

**Table S3 % of human NORs associated with nucleoli in human/mouse monochromosomal hybrid cells**

| Cell line | Number of nucleoli with a chromosome colocalized | Number of nucleoli without a chromosome colocalization | Total number of analyzed nucleoli | % of nucleoli with colocalized chromosomes |
| --- | --- | --- | --- | --- |
| A9(#13) 89-2 (chr13) | 134 | 183 | 317 | 42% |
| A9hygro14 (chr14) | 152 | 27 | 179 | 85% |
| A9(Neo15)-3 (chr15) | 98 | 30 | 128 | 77% |
| A9 #21-16 (chr21) | 130 | 154 | 284 | 46% |
| A9#22(γ2) (chr22γ2) | 58 | 85 | 143 | 41% |
| A9HyTK-22 (chr22TK) | 42 | 99 | 141 | 30% |
| A9KM10-4 (chr 4) | 28 | 102 | 130 | 21% |
| A9 48-4 (chr 18) | 43 | 131 | 174 | 24% |

**Table S4 % of human NORs associated with nucleoli in human/mouse monochromosomal hybrid cells A9(#13) 89-2 (chr13) after reactivation of rDNA transcription**

| Cell line |  | Number of nucleoli with a chromosome colocalized | Number of nucleoli without a chromosome colocalization | Total number of analyzed nucleoli | % of nucleoli with colocalized chromosomes |
| --- | --- | --- | --- | --- | --- |
| A9(#13) 89-2 (chr13) | Exp. 1 | 36 | 50 | 86 | 40 |
|  | Exp. 2 | 66 | 82 | 148 | 44 |
|  | Exp. 3 | 42 | 55 | 97 | 43 |
|  | Total | 144 | 187 | 331 | 42 |
| A9(#13) 89-2 (chr13) + pLVTHM TAFs | Exp. 1 | 93 | 54 | 147 | 63 |
|  | Exp. 2 | 76 | 60 | 136 | 55 |
|  | Exp. 3 | 73 | 49 | 122 | 59 |
|  | Total | 242 | 163 | 405 | 59 |

**Table S5 Measurement of the nucleolus area within the nucleus**

| Cell line | Average square of nucleus (μm^2^) | Average square of nucleolus (μm^2^) | Average ratio square of nucleolus/ square of nucleolus |
| --- | --- | --- | --- |
| A9(#13) 89-2 (chr13) | 72.45 | 15.94 | 0.22 |
| A9hygro14 (chr14) | 54.98 | 10.48 | 0.194 |

**Table S6** **% of HACs associated with nucleoli in human HT1080 cells**

| Construct  name | Number of nucleoli with colocalized HAC | Number of nucleoli without HAC colocalization | Total number of analyzed nucleoli | % of nucleoli with colocalized HAC | P value |
| --- | --- | --- | --- | --- | --- |
| TAR1 (rDNA) | 201 | 90 | 291 | **69%** | **< 0.05** |
| TAR2 (45S) | 174 | 105 | 279 | **62%** | **< 0.05** |
| TAR3 (IGS) | 224 | 141 | 365 | **61%** | **< 0.05** |
| IGS-Δ | 68 | 98 | 166 | 41% | 0.628 |
| TAR4 (PJ) | 139 | 178 | 317 | 44% | 0.170 |
| TAR5 (DJ) | 253 | 103 | 356 | **71%** | **< 0.05** |
| GFP | 189 | 266 | 455 | **42%** |  |

**Table S7 % of HACs associated with nucleoli in dox+ and dox-media**

| Conditions | Number of nucleoli with a  colocalized HAC | Number of nucleoli without a HAC colocalization | Total number of analyzed nucleoli | % of nucleoli with a colocalized HAC |
| --- | --- | --- | --- | --- |
| HAC/GFP (dox+) | 173 | 200 | 373 | 46 |
| HAC/GFP (dox-) | 162 | 197 | 359 | 45 |
| HAC/GFP+ tetR-tTA^VP64^ (dox+) | 154 | 177 | 331 | 47 |
| HAC/GFP+ tetR-tTA^VP64^ (dox-) | 107 | 206 | 313 | 34 |
| HAC/rDNA (dox+) | 185 | 88 | 273 | 68 |
| HAC/rDNA (dox-) | 208 | 92 | 300 | 69 |
| HAC/rDNA+ tetR-tTA^VP64^ (dox+) | 229 | 99 | 328 | 70 |
| HAC/rDNA+ tetR-tTA^VP64^ (dox-) | 155 | 169 | 324 | 48 |
| HAC/DJ (dox+) | 249 | 110 | 359 | 69 |
| HAC/DJ (dox-) | 178 | 89 | 267 | 67 |
| HAC/DJ+ tetR-tTA^VP64^ (dox+) | 174 | 92 | 266 | 65 |
| HAC/DJ+ tetR-tTA^VP64^ (dox-) | 117 | 120 | 237 | 49 |

**Table S8 % of HAC localized on the nucleoli periphery versus nucleoli stroma in dox+ and dox-media**

| Conditions | Number of HACs localized on nucleoli periphery | Number of HACs localized on nucleoli stroma | Total number of analyzed nucleoli | % of nucleoli with HACs localized on nucleoli periphery |
| --- | --- | --- | --- | --- |
| HAC/GFP+ tetR-tTA^VP64^ (dox+) | 180 | 40 | 220 | 82 |
| HAC/GFP+ tetR-tTA^VP64^ (dox-) | 162 | 31 | 193 | 84 |
| HAC/rDNA+ tetR-tTA^VP64^ (dox+) | 147 | 28 | 175 | 84 |
| HAC/rDNA+ tetR-tTA^VP64^ (dox-) | 177 | 44 | 221 | 80 |

**Table S9**

**(**fttp://ncbi.nlm.nih.gov/pub/shabalin/DJ_Region/Supplementary Table S9)

**Table S10** **A list of conserved between human and chimpanzee and experimentally validated TF sites in the 92.5-94.5 kb region**

Stable ID: ENSM00000419675

Location: GL000220.1:93039-93055

Binding matrix: ENSPFM0042

Transcription factors **CTCF**

Experimentally verified in Hu. Fe., A549, GM12878, K562, SK-N., MCF-7, keratinocyte, bipolar neuron, cardiac muscle, HepG2, MM.1S, IMR-90, H1-hESC_3, hepatocyte, A673, transverse colon, astrocyte spinal cord, foreskin fibroblast_1

Stable ID: ENSM00197656300

Location: GL000220.1:93502-93515

Binding matrix: ENSPFM0085

Transcription factors **EBF1**

Experimentally verified in GM12878

Stable ID: ENSM00000159588

Location: GL000220.1:93691-93707

Binding matrix: ENSPFM0042

Transcription factors **CTCF**

Experimentally verified in Hu. Fe., HeLa-S3, A549, pancreas_2, GM12878, K562, SK-N., MCF-7, NHLF, keratinocyte, bipolar neuron, cardiac muscle, B, HepG2, MM.1S, IMR-90, PC-9, CD14+ monocyte_1, HUVEC, H1-hESC_3, osteoblast, HSMM, PC-3, HCT116, hepatocyte, A673, mammary epithelial_1, myotube, astrocyte, dermal fibroblast, transverse colon, astrocyte cerebellum, DND-41, astrocyte spinal cord, foreskin fibroblast_1

Stable ID: ENSM01052209394

Location: GL000220.1:93723-93736

Binding matrix: ENSPFM0487

Transcription factors: **RXRG, RXRA, RORB**

Experimentally verified in HepG

Stable ID: ENSM00524452267

Location: GL000220.1:93747-93758

Binding matrix: ENSPFM0427

Transcription factors **NRF1**

Experimentally verified in K562, HepG2

Stable ID: ENSM01051890022

Location: GL000220.1:93747-93760

Binding matrix: ENSPFM0086

Transcription factors **EGR1, EGR2, EGR4, EGR3**

Experimentally verified in K562

Stable ID: ENSM00100257483

Location: GL000220.1:93817-93827

Binding matrix: ENSPFM0357

Transcription factors **KLF14, SP4, KLF16, SP8, SP3, SP1**

Experimentally verified in H1-hESC_3

Stable ID: ENSM00040818633

Location: GL000220.1:93879-93889

Binding matrix: ENSPFM0186

Transcription factors **FOXB1, FOXC1, FOXA1, FOXC2**

Experimentally verified in HepG2

Stable ID: ENSM00523520434

Location: GL000220.1:93882-93893

Binding matrix: ENSPFM0366

Transcription factors **MEF2A, MEF2D, MEF2B**

Experimentally verified in GM12878

Stable ID: ENSM00096422097

Location: GL000220.1:93900-93911

Binding matrix: ENSPFM0350

Transcription factors **IRF2**

Experimentally verified in K562

Stable ID: ENSM00145521040

Location: GL000220.1:93919-93932

Binding matrix: ENSPFM0290

Transcription factors **HNF4A, RXRG, NR2F6, NR2F1, NR2C2, RXRA, RXRB**

Experimentally verified in HepG2

Stable ID: ENSM00524154916

Location: GL000220.1:93931-93941

Binding matrix: ENSPFM0614

Transcription factors **YY2, YY1**

Experimentally verified in K56

Stable ID: ENSM00206651778

Location: GL000220.1:93984-93998

Binding matrix: ENSPFM0363

Transcription factors **MAFF, MAFG, MAFK**

Experimentally verified in HeLa-S3, A549, GM12878, K562, HepG2, IMR-90, H1-hESC_3

Stable ID: ENSM00184630099

Location: GL000220.1:94070-94080

Binding matrix: ENSPFM0614

Transcription factors **YY2, YY1**

Experimentally verified in GM12892, SK-N-SH_RA, GM12878, H1-hESC_3

Stable ID: ENSM00101682473

Location: GL000220.1:94136-94147

Binding matrix: ENSPFM0364

Transcription factors **MAFK, NRL**

Experimentally verified in HeLa-S3, A549, GM12878, K562, HepG2, IMR-90, H1-hESC_3

Stable ID: ENSM00522999631

Location: GL000220.1:94332-94345

Binding matrix: ENSPFM0392

Transcription factors **MYBL1::ELF1**

Experimentally verified in K562

Stable ID: ENSM00526279279

Location: GL000220.1:94382-94396

Binding matrix: ENSPFM0191

Transcription factors **FOXO1::ELF1, FOXJ2::ELF1, FOXJ3::ELF1**

Experimentally verified in K562

Stable ID: ENSM00524689175

Location: GL000220.1:94395-94416

Binding matrix: ENSPFM0305

Transcription factors **HOXB2::ELF1**

Experimentally verified in K562

**Table S11 The most common motifs that were found at the proximal end of the DJ region by the DME-X program**

ID DME20

SEQ GGGAGG

FGCOUNT=140

INFO=2.21389

SCORE=13.2833

ID DME28

SEQ AGGCTG

FGCOUNT=129

INFO=2.11006

SCORE=12.6604

ID DME29

SEQ CTGGGA

FGCOUNT=127

INFO=2.11006

SCORE=12.6604

ID DME23

SEQ CAGAGA

FGCOUNT=137

INFO=2.05693

SCORE=12.3416

ID DME3

SEQ AGAAAG

FGCOUNT=291

INFO=1.99122

SCORE=11.9473

ID DME5

SEQ GAAAGA

FGCOUNT=257

INFO=1.99122

SCORE=11.9473

ID DME13

SEQ GAAGAA

FGCOUNT=167

INFO=1.99122

SCORE=11.9473

ID DME15

SEQ AGAGAA

FGCOUNT=164

INFO=1.99122

SCORE=11.9473

ID DME18

SEQ AGAAGA

FGCOUNT=158

INFO=1.99122

SCORE=11.9473

ID DME19

SEQ GAGAAA

FGCOUNT=157

INFO=1.99122

SCORE=11.9473

ID DME22

SEQ AGGAAA

FGCOUNT=143

INFO=1.99122

SCORE=11.9473

ID DME24

SEQ AAGAAG

FGCOUNT=139

INFO=1.99122

SCORE=11.9473

ID DME26

SEQ AAGAGA

FGCOUNT=138

INFO=1.99122

SCORE=11.9473

ID DME14

SEQ CAGAAA

FGCOUNT=167

INFO=1.98271

SCORE=11.8963

ID DME21

SEQ ACAGAA

FGCOUNT=146

INFO=1.98271

SCORE=11.8963

ID DME25

SEQ AAACAG

FGCOUNT=139

INFO=1.98271

SCORE=11.8963

ID DME27

SEQ AAGCAA

FGCOUNT=138

INFO=1.98271

SCORE=11.8963

ID DME30

SEQ TGGAAA

FGCOUNT=135

INFO=1.97012

SCORE=11.8207

ID DME1

SEQ AAGAAA

FGCOUNT=427

INFO=1.91699

SCORE=11.502

ID DME2

SEQ AAAGAA

FGCOUNT=390

INFO=1.91699

SCORE=11.502
